# Rapid and Repeated Climate Adaptation Involving Chromosome Inversions following Invasion of an Insect

**DOI:** 10.1093/molbev/msae044

**Published:** 2024-02-24

**Authors:** Li-Jun Ma, Li-Jun Cao, Jin-Cui Chen, Meng-Qing Tang, Wei Song, Fang-Yuan Yang, Xiu-Jing Shen, Ya-Jing Ren, Qiong Yang, Hu Li, Ary Anthony Hoffmann, Shu-Jun Wei

**Affiliations:** Institute of Plant Protection, Beijing Academy of Agriculture and Forestry Sciences, Beijing 100097, China; Institute of Plant Protection, Beijing Academy of Agriculture and Forestry Sciences, Beijing 100097, China; Institute of Plant Protection, Beijing Academy of Agriculture and Forestry Sciences, Beijing 100097, China; Institute of Plant Protection, Beijing Academy of Agriculture and Forestry Sciences, Beijing 100097, China; Department of Entomology and MOA Key Lab of Pest Monitoring and Green Management, College of Plant Protection, China Agricultural University, Beijing 100193, China; Institute of Plant Protection, Beijing Academy of Agriculture and Forestry Sciences, Beijing 100097, China; Institute of Plant Protection, Beijing Academy of Agriculture and Forestry Sciences, Beijing 100097, China; Institute of Plant Protection, Beijing Academy of Agriculture and Forestry Sciences, Beijing 100097, China; Institute of Plant Protection, Beijing Academy of Agriculture and Forestry Sciences, Beijing 100097, China; Department of Entomology and MOA Key Lab of Pest Monitoring and Green Management, College of Plant Protection, China Agricultural University, Beijing 100193, China; Bio21 Institute, School of BioSciences, University of Melbourne, Parkville, Victoria 3010, Australia; Department of Entomology and MOA Key Lab of Pest Monitoring and Green Management, College of Plant Protection, China Agricultural University, Beijing 100193, China; Bio21 Institute, School of BioSciences, University of Melbourne, Parkville, Victoria 3010, Australia; Institute of Plant Protection, Beijing Academy of Agriculture and Forestry Sciences, Beijing 100097, China

**Keywords:** climate change, population genomics, local adaption, phenotypic variation, structure variation

## Abstract

Following invasion, insects can become adapted to conditions experienced in their invasive range, but there are few studies on the speed of adaptation and its genomic basis. Here, we examine a small insect pest, *Thrips palmi*, following its contemporary range expansion across a sharp climate gradient from the subtropics to temperate areas. We first found a geographically associated population genetic structure and inferred a stepping-stone dispersal pattern in this pest from the open fields of southern China to greenhouse environments of northern regions, with limited gene flow after colonization. In common garden experiments, both the field and greenhouse groups exhibited clinal patterns in thermal tolerance as measured by critical thermal maximum (CT_max_) closely linked with latitude and temperature variables. A selection experiment reinforced the evolutionary potential of CT_max_ with an estimated *h*^2^ of 6.8% for the trait. We identified 3 inversions in the genome that were closely associated with CT_max_, accounting for 49.9%, 19.6%, and 8.6% of the variance in CT_max_ among populations. Other genomic variations in CT_max_ outside the inversion region were specific to certain populations but functionally conserved. These findings highlight rapid adaptation to CT_max_ in both open field and greenhouse populations and reiterate the importance of inversions behaving as large-effect alleles in climate adaptation.

## Introduction

Climate is one of the key environmental factors determining the distribution and population density of insect species (Hewitt 2004; Clusella-Trullas and Nielsen 2020; Ma et al. 2020; Marshall et al. 2020). In the Anthropocene, species are facing unprecedented rapid climate changes linked to global warming, range expansion, and human-altered environments (Chown et al. 2015; Renault et al. 2018; Waters and Turner 2022). Understanding the ability of species to survive climate changes and underlying mechanisms are key issues in ecology and evolution. Although plasticity may buffer organisms in novel climates, a growing number of studies have shown that adaptation to local conditions plays important roles in species facing climate-related selective pressures (Hoffmann and Sgrò 2011; Hoffmann 2017; Dai et al. 2023). Biological invasion provides particularly good opportunities to understand the pace of such adaptive processes and their underlying mechanisms in nature (Lee 2002; Colautti and Barrett 2013; Chown et al. 2015; Battlay et al. 2023). Invasive species often encounter new environmental conditions, and these can include new climate conditions as invaders spread over a wide geographic range in a short time period. However, to date, there are few studies on climate adaptation in invaders particularly in insect species (Rezende et al. 2010; Hoffmann 2017; Pelissie et al. 2018; McCulloch and Waters 2023).

The gold standard for detecting local adaptation includes common garden and reciprocal transplant experiments (Villemereuil et al. 2016; Johnson et al. 2022). However, these tests remain difficult in many species, including insects, because phenotypic measurements can be affected by environmental conditions and inbreeding when rearing populations from different locations, and because it can be difficult to simulate environmental conditions relevant to past selection events (Hoffmann and Sgrò 2018). An increasing number of insect studies are instead using population genomic approaches to study local adaptation by associating genotypic data from multiple populations with environmental variables (Mérel et al. 2021; North et al. 2021; Parvizi et al. 2023). Population genomic approaches can detect signals of local adaptation and help to identify the genetic basis of adaptive traits from genomic variation (Jensen et al. 2016; Bernatchez et al. 2023; McCulloch and Waters 2023). However, these methods can lead to false adaptive signals, even when there are methodological attempts to correct the influence of genetic drift, demographic history, and other population processes (Andrews and Luikart 2014; Lotterhos and Whitlock 2015; Francois et al. 2016; Booker et al. 2020; Hoffmann et al. 2021).

Climatic adaptation is expected to be typically polygenic, involving multiple sets of genes leading to the same phenotypic change (Barghi et al. 2020), as documented in recent studies (Csilléry et al. 2018; Healy et al. 2018; Yang et al. 2022). Moreover, climatic adaptation can involve genes tied up in inversion polymorphisms that inhibit recombination and make the identification of genetic markers linked to genes under selection difficult (Hoffmann and Rieseberg 2008; Galludo et al. 2018; Kapun and Flatt 2019). These complications and the potentially pervasive effects of genetic backgrounds challenge the meaningful study of climatic adaptation using only population genomic approaches (Hoffmann et al. 2021). Instead, genomic evidence, phenotypic data, and their combinations need to be integrated when studying rapid adaptative evolution to climatic stresses following invasion into new areas, an approach that has been widely used in plant species (Colautti and Barrett 2013; Helliwell et al. 2018; van Boheemen et al. 2019; Battlay et al. 2023), but less in insects where variation is measured either at the phenotypic or genomic levels (Goubert et al. 2017; Waldvogel et al. 2018; Sherpa et al. 2022; Dai et al. 2023), but rarely combined (Sherpa et al. 2022).

*Thrips* species represent small insects from the order Thysanoptera. Several thrips cause serious damage to crops worldwide (Morse and Hoddle 2006; Mound et al. 2022). The melon thrips, *Thrips palmi*, was first described in tropical regions of Sumatra in Indonesia. Early reports of *T. palmi* were exclusively from southeastern and southern Asian countries. The species was introduced into several new areas and has become established during the second half of the 20th century across Southeast Asia, South America, Australia, and West Africa (Cannon et al. 2007). In China, *T. palmi* was first found in southern provinces in the 1970s and has gradually spread northward in the past 50 yr (Cao et al. 2019; Gao et al. 2021). Climatic conditions are thought to limit the distribution of *T. palmi* (Mcdonald et al. 1999). This pest cannot survive in the area above 34°N latitude in natural conditions (Sakimura et al. 1986). However, *T. palmi* expanded to temperate regions in Japan and China in greenhouses where it is one of the most severe pests of vegetables (Gao et al. 2021).

The shift of geographical distribution from tropical to warm areas may pose strong temperature pressures to populations of *T. palmi*. Although the greenhouse environment can help *T. palmi* to overcome the cold winter, it also imposes frequent high-temperature stress on populations when compared to those in an open field (Shamshiri et al. 2018). A microsatellite study has previously shown that the genetic structure of *T. palmi* differs between populations collected from field and greenhouse environments, with temperature-related climatic variables linked to genetic variants (Cao et al. 2019). The well-recorded dispersal history, biology, and ecology of this pest make it a useful species to investigate climatic adaptation following invasion.

In this study, we hypothesized that populations have become adapted to local climates across the distribution range of *T. palmi* since invasion. We undertook whole-genome resequencing of samples from across its geographic range in China while also testing the tolerance of populations to high and low temperatures in a common garden design. We found parallel evolution of heat but not cold stress tolerance in 2 population groups linked to local temperature and also evidence of genomic changes that could be interpreted in terms of thermal adaptation. Our study provides insights into the genomic basis of rapid evolution of an invasive insect during range expansion, reiterates the importance of chromosome inversions in climate adaptation, but also highlights the challenges in linking genomic approaches to adaptive phenotypic changes in traits.

## Results

### Population Genomic Variation and Demographic History

**Fig. 1.**
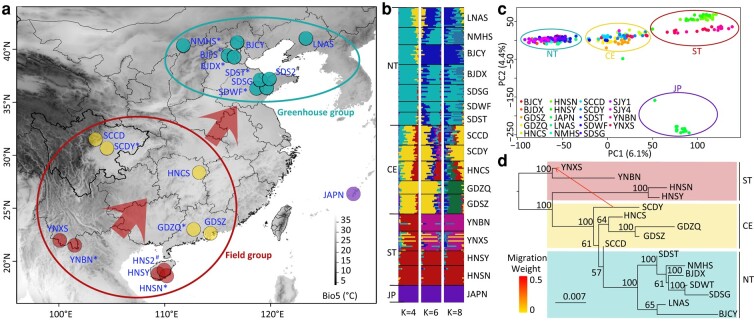
Population genetic structure of *T. palmi*. a) Map of sampled populations in the study. Arrows show the dispersal routes as inferred by demographic analysis and historical records. Eleven populations were used for thermal stress tests, 8 of which had CT_max_ data as shown by * following the population code and 3 of which did not have genomic data as shown by ^#^ (see supplementary table S1, Supplementary Material online, for detailed information on the populations). b) Model-based population assignment using ADMIXTURE with *K* = 4, 6, and 8. The height of each segment represents the proportion of the individual's genome derived from inferred ancestral lineages. Four genetic groups (JP, Japan group; ST, southern group; Ce, central group; NT, northern group) were shown. c) Population genetic structure of the *T. palmi* populations inferred from PCA. For population codes, refer to Table 1. Four population groups are identified. The percent variance accounted for by each PC is indicated in brackets. d) Structure inferred by TreeMix for *T. palmi* populations in China using Yunnan populations as outgroups, allowing 1 migration event. Migration occurred between SCDY and YNXS. See the color key for migration weight of the arrow.

To examine population genomic variation and the demographic history of *T. palmi*, we conducted whole-genome resequencing for 249 individuals from 17 geographical populations sampled across its natural distribution of China and Japan, as well as a heat stress–selected population and its control population in the laboratory (Fig. 1a; supplementary table S1, Supplementary Material online). On average, about 86% of the clean reads were aligned to the reference genome, with an average depth of 45× (supplementary table S2, Supplementary Material online). A total of 930,895 single nucleotide polymorphisms (SNPs) remained after filtering.

Individuals were optimally assigned into 8 genetic clusters (Fig. 1b). The Japanese population was composed of a unique genetic cluster. The other 7 clusters were mainly distributed in southern, central, and northern population groups. The southern and central groups correspond to areas where *T. palmi* can overwinter in open fields and are referred to as the field group; the northern group corresponds to areas where *T. palmi* survive winter climates in greenhouses and are referred to as the greenhouse group (Fig. 1b and c). There is a significant correlation between genetic and geographical distances among all populations (Mantel’s test, *r* = 0.594, *P* < 0.0001), suggesting a stepping-stone dispersal at the larger scale in China (supplementary fig. S1, Supplementary Material online). However, when groups were analyzed separately, a significant correlation was found in the field group (*r* = 0.308, *P* = 0.026), but not in the greenhouse group (*r* = −0.030, *P* = 0.459). KimTree analysis showed that the southern group was ancestral to the other groups, reflecting a northward dispersal of these populations (supplementary fig. S3A, Supplementary Material online). The northward dispersal was further supported by population genetic diversity analysis, where nucleotide diversity declined from southern to northern populations generally, and 2 southwestern populations, YNXS (*π* = 0.0063) and YNBN (*π* = 0.0064), had the highest nucleotide diversity (supplementary table S3, Supplementary Material online).

There was a high level of gene flow associated with the range expansion, since most individuals from the field group had high levels of genetic admixture (supplementary fig. S2, Supplementary Material online). However, TreeMix analysis revealed that adding an optimal migration event from SCDY to YNXS only slightly increased the explained variance (mean 93.37%) among populations compared to a strict drift model (mean 92.07%; Fig. 1d; supplementary fig. S4, Supplementary Material online). This suggests limited gene flow among *T. palmi* populations after the expansion had occurred.

### Stronger Effects of Climate Than Geography on Genomic Variation

We tested whether geography and climate variables can explain the observed genomic variation among populations of *T. palmi*. Multivariate redundancy analysis (RDA) showed that 34.52% (all populations), 39.75% (field group), and 34.85% (greenhouse group) genetic variations can be explained by the combined effects of neutral genetic structure, climatic and geographical variables, and their interactions. After controlling for neutral genetic variation, climate (all: 18.66%; field: 20.40%; greenhouse: 17.7%) explained a higher proportion of the genetic variance than geography (all: 5.72%; field: 3.37%; greenhouse: 0; Table 1), highlighting the importance of climatic variables in explaining the genomic variation.

**Table 1:** Results of RDA models testing the impact of climate, genetic structure deduced from neutral markers, and geography on genetic differences among populations

Population	RDA model	Variance	*R* ^2^	*P*	Proportion of explainable variance (%)	Proportion of total variance (%)
All populations	Full model	222,800	0.2566	0.001	100.00	34.52
	Only climate	41,579	0.0479	0.001	18.66	6.44
	Only structure	48,749	0.0561	0.001	21.88	7.55
	Only geography	12,754	0.0147	0.001	5.72	1.98
	Confounded climate/structure/geography	119,718			53.73	18.55
	Total unexplained	422,609				65.48
	Total variance	645,409				100.00
Field group	Full model	254,774	0.2844	0.001	100.00	39.75
	Only climate	51,978	0.0580	0.001	20.40	8.11
	Only structure	55,925	0.0624	0.001	21.95	8.72
	Only geography	8580	0.0096	0.003	3.37	1.34
	Confounded climate/structure/geography	138,291			54.28	21.57
	Total unexplained	386,209				60.25
	Total variance	640,983				100.00
Greenhouse group	Full model	181,533	0.2584	0.001	100.00	34.85
	Only climate	32,142	0.0458	0.001	17.71	6.17
	Only structure	80,852	0.1151	0.001	44.54	15.52
	Only geography	0	0	0	0	0
	Confounded climate/structure/geography	68,539			37.76	13.16
	Total unexplained	339,404				65.15
	Total variance	520,937				1.00

### Repeated Phenotypic Adaptation to Climate and Latitude

**Fig. 2.**
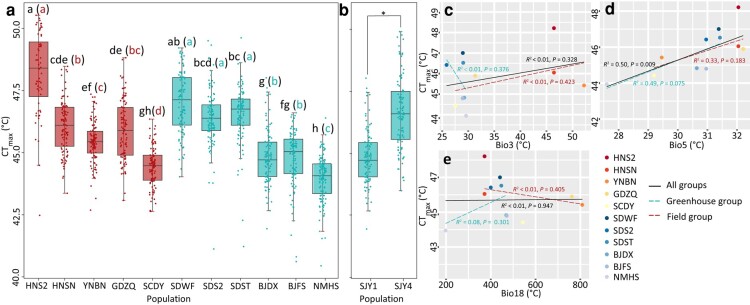
The heat tolerance of *T. palmi* populations. a) CT_max_ value of the tested individuals from each population. Box plot colors match genetic groups: the first five populations are from the field group and the last six populationa are from the greenhouse group. The lowercase on the boxplot indicates the result of multiple comparisons between populations. Lowercase colors represent different groups: black for both groups; red for the field group; and blue for the greenhouse group. b) CT_max_ value of BJDX population before (SJY1) and after heat stress selection (SJY4). c to e) Correlation between CT_max_ and climatic data and their importance in identifying genomic variants. Linear regressions between drop temperature (measure of CT_max_) and c) isothermality (bio3), d) maximum temperature of the warmest month (bio5), and e) precipitation of the warmest quarter (bio18). Red dotted line and blue dotted line represent the trend line of linear regression for the field group and the greenhouse group.

The northward range expansion of *T. palmi* was accompanied by changes in local climate, which we expected to be related to heat stress in the southern and greenhouse populations and cold stress in the open field populations. Thus, heat and cold stress tolerance were measured by testing critical thermal maximum (CT_max_) and chill coma recovery time (CCRT), respectively, in 11 geographical populations using common garden experiments (Fig. 1a). Female adults had a significantly higher heat tolerance than male adults (Mann–Whitney *U* test, *P* < 0.01), while no significant difference was found between female and male adults in cold tolerance (*P* = 0.064; supplementary fig. S5, Supplementary Material online). Thus, 2-d-old female adults were used to test for the phenotypic differences among populations. There was a significant difference in CT_max_ and CCRT among populations when all populations, field and greenhouse groups were analyzed separately (Kruskal–Wallis test, df = 10, *P* < 0.025; supplementary table S4, Supplementary Material online). Populations collected from Hainan Island of the southern group and those from Shandong province of the greenhouse group had the highest CT_max_ values, while population NMHS from the northern area of greenhouse group had the lowest CT_max_, followed by SCDY from the northern area of the field group. Among populations from the field group, CT_max_ declined from low- to high-latitude regions (Fig. 2a). The clinal pattern with latitude was also found among populations from the greenhouse group (Fig. 2a; supplementary table S5, Supplementary Material online), indicating clinal and parallel evolution of heat stress tolerance in field and greenhouse populations in China. However, there was no obvious geographical pattern between changes in CCRT among populations and latitude (supplementary fig. S6 and table S6, Supplementary Material online).

### Rapidly Increased Heat Stress Tolerance in an Artificially Selected Population

To validate the capacity and speed of evolutionary heat stress adaptation in *T. palmi*, population BJDX collected from northern China was selected under heat stress in the laboratory. About 50% of individuals surviving a dynamically increasing heat stress were selected at the F_0_ to F_3_ generations. The CT_max_ of the F_4_ generation (SJY4) was significantly higher than that of the F_0_ generation (SJY1; Mann–Whitney *U* test, *P* < 0.01; Fig. 2b), indicating the potential for rapid evolution of *T. palmi* under heat stress. The realized heritability (*h*^2^) of CT_max_ was 0.068.

### Association between Phenotypic and Climatic Data

To examine whether population phenotypes are associated with climate of the locations where they were collected, we conducted an association analysis (supplementary tables S7 and S8, Supplementary Material online). For the 3 remaining bioclimatic variables after removing correlated variables (Fig. 2c to e), there was a relatively strong association between CT_max_ of the populations and the temperature-related variable bio5 (maximum temperature of the warmest month; *R*^2^ = 0.50, 0.33, 0.49, *P* = 0.009, 0.183, 0.075 for all populations, field group, and greenhouse group, respectively, linear regression), but a weak association between CT_max_ of the populations and another temperature-related variable bio3 [isothermality (bio2/bio7) (×100); *R*^2^ < 0.01, *P* > 0.328], as well as a precipitation-related variable bio18 (precipitation of warmest quarter; *R*^2^ < 0.08, *P* > 0.301). The correlation between CCRT and the tested variables is weak (supplementary table S9, Supplementary Material online). In both the field and greenhouse groups, CT_max_ and bio5 had similar importance in accounting for genomic variation as shown in a gradient forest (GF) analysis (supplementary fig. S7, Supplementary Material online).

### Chromosome Inversions Potentially Related to Phenotypic Variation

**Fig. 3.**
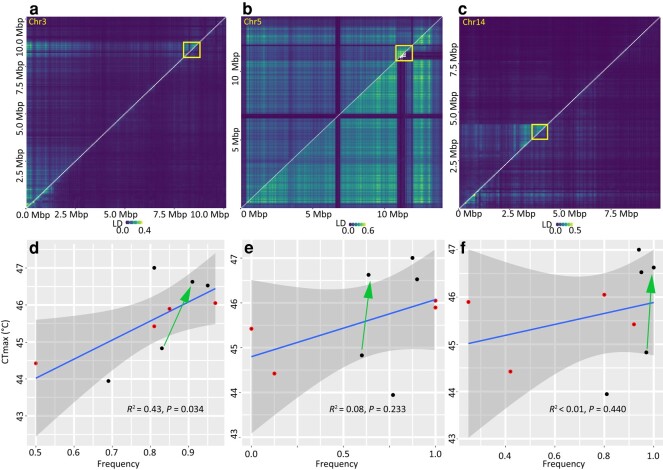
Structural variations in populations of *T. palmi*. Heatmap of pairwise LD for 50-kb windows for chr3 a), chr5 b), and chr14 c); the upper triangle shows the LD values for all individuals, while the lower triangle shows the LD values when individuals carrying the homozygous genotype of only 1 allele were kept; the box shows the inversion region. Correlation between CT_max_ values and allele frequency of genomic structural variants in chr3 d), chr5 e), and chr14 f). Two points at the start and end of green arrows indicate 2 populations before and after heat stress selection, respectively; the red point indicates population collected from open field environment, while the black point indicates populations collected from greenhouse environment.

To examine the genetic basis of heat stress adaptation in *T. palmi*, we first considered structural variants in populations. Three chromosome inversions were identified on chromosomes chr3, chr5, and chr14, with their length being 667, 1,130, and 764.6 kb, respectively (supplementary fig. S8A to C, Supplementary Material online). Individuals with the heterozygous genotype of the inverted region are expected to have higher observed heterozygosity of SNPs than 2 homozygous genotypes due to limited genetic exchange between inverted and noninverted regions. When we plot the observed heterozygosity of each individual against the first component of the principal component analysis (PCA; PC1) using SNPs in the inversion regions, 3 clusters of individuals were found, corresponding to 2 homozygous genotypes (2 sides) and a heterozygous genotype (middle; supplementary fig. S8D to F). As expected, due to limited genetic exchange, the inverted regions had a higher linkage disequilibrium (LD) value than the other regions when all individuals were analyzed (upper triangle in Fig. 3a to c). When we only considered individuals with the homozygous genotype, the LD was reduced (lower triangle in Fig. 3a to c). These LD analyses therefore further support the presence of chromosome inversions in the absence of cytological data.

We conducted an exploratory association analysis between CT_max_ and the frequency of putative inversions using 4 field and 4 greenhouse populations as well as the 2 populations before and after heat stress selection. A positive association was found between CT_max_ values of populations and the frequency of the putative inversion in chr3 (linear regression, *R*^2^ = 0.43, *P* = 0.034; Fig. 3d), but the association was weak for the putative inversions in chr5 and chr14 (linear regression, *R*^2^ = 0.08 and *P* = 0.233 for chr5; *R*^2^ < 0.01 and *P* = 0.440 for chr14; Fig. 3e and f). In the laboratory population, the frequency of the inversion in chr3 increased from 83.33% to 90.91% after selection, and in chr5, the inversion increased from 60.00% to 63.64% after selection, both linked to CT_max_ increases after heat stress selection (green arrows in Fig. 3d and e).

There are 16, 86, and 66 genes in the inverted regions of chr3, chr5, and chr14, respectively. Five genes in the inverted region of chr3 are related to phospholipid metabolic process, 17 genes in the inverted region of chr14 are related to energy metabolism and oxidation–reduction, and 5 genes in this region in chr5 are related to oxidation–reduction (supplementary appendix S1, Supplementary Material online).

### Different Outlier Genes with Similar Functions Potentially Related to Climatic Adaptation

Outlier SNPs potentially related to heat stress tolerance were scanned based on population differentiation or variation among individuals with different levels of heat stress tolerance. To reduce the influence of population history on the genome scan, 2 populations (BJCY and HNCS) having distinct patterns of effective population size (*N*_e_) from the others were excluded (supplementary fig. S3B, Supplementary Material online). For overall population comparisons, we examined differentiation across the genome using a *k*-nearest neighbor (kNN) analysis for the field and greenhouse groups separately (supplementary fig. S9, Supplementary Material online). In pairwise comparisons, *F*_ST_ was calculated between populations with high and low CT_max_ values, between the laboratory-selected population and its control population, and between 10 individuals with high CT_max_ and 10 individuals with low CT_max_ values within a population (supplementary appendix S2 and fig. S10, Supplementary Material online). We found outlier genes associated with functions in cytoskeletal organization (actin and microtube binding genes) and oxidation–reduction as well as heat stress response (HSP70, HSP90, and DnaJ) in all 10 analyses except for HSP in the laboratory-selected population (supplementary table S9, Supplementary Material online). These findings point to the same pathways being under selection due to heat stress measured in different contexts. However, although the same gene functions appeared in these analyses, specific genes were rarely shared between them (supplementary table S10, Supplementary Material online).

We also conducted genotype–phenotype/climate association analysis using a univariate latent factor linear mixed model (LFMM). Genes functioning in cytoskeletal organization, oxidation–reduction, and heat stress response were identified in most analyses (supplementary appendix S3, table S9, and fig. S11, Supplementary Material online). In 4 field populations with phenotypic data, 67.2% of outlier genes were shared between LFMM analysis based on CT_max_ and bio5 (maximum temperature of the warmest month); in the same analysis for 4 greenhouse populations with phenotypic data, the proportion was 95.5%. However, we found that the specific outlier-associated genes differed in LFMM analysis between genomic variation and bio5 when several populations without CT_max_ values were added (supplementary table S11, Supplementary Material online), indicating the sensitivity of this analysis to the data set used.

We compared genes identified as outliers and genes on the inverted genomic regions. Of the 16, 87, and 66 genes located on inverted regions of chr3, chr5, and chr14, 8, 77, and 11 were identified as outliers in at least one of the genome scan analyses, respectively (supplementary tables S12 and S13, Supplementary Material online). All 5 genes related to oxidation–reduction on the inverted regions of chr5 were identified as outliers.

## Discussion

This study tested the adaptive variation of an invertebrate insect that expanded its distribution range from subtropical to temperate regions and then to greenhouse environments in areas where it could not survive the winter in open fields (McDonald et al. 2000). While measuring phenotypic variation provides direct evidence for local adaptation, this approach is challenging for many species due to difficulties in field collection, rearing, testing trait selection, and simulating environments (Hoffmann and Sgrò 2018). Population genomic approaches offer alternative ways to detect climatic adaptation in species beyond phenotypic measurements, as well as providing a way to understand the genetic basis of trait evolution (Pelissie et al. 2018). However, genomic variation can be shaped by neutral as well as adaptive evolutionary processes, and it can be influenced by the genetic architecture of traits, challenging the robustness of purely genomic comparisons (Francois et al. 2016). By measuring genetic and phenotypic variation in species using a combination of population genomics, common garden experiments, and artificial selection, a deeper understanding of the pace of adaptive evolution under rapid environmental changes can be obtained (Villemereuil et al. 2016; McCulloch and Waters 2023).

Studies using multiple lines of evidence are being increasingly used to identify rapid local adaptation in invasive plants (Colautti and Barrett 2013; Helliwell et al. 2018; van Boheemen et al. 2019). However, these types of studies remain uncommon in insect invaders. Phenotypic and genetic lines of evidence have been used to investigate climate adaptation and its genetic basis in *Drosophila subobscura* following its recent invasion into the Americas (Galludo et al. 2018). Genomic data have also been used extensively to investigate patterns of invasion in insect pests and disease vectors (Sherpa and Després 2021). In pest species like the fruit fly *Bactrocera dorsalis* (Zhang et al. 2023) and the small hive beetle *Aethina tumida* (Liu et al. 2021), genomic data collected across multiple populations have been used to identify genetic markers associated with environmental factors, and in at least 1 case, the impact of identified genes on climate adaptation has been investigated through altering the expression of a candidate gene with RNAi (Zhang et al. 2023). While these studies can point to local adaptation following in the invasion process, a combination of genomic analyses and phenotypic data collected under common garden conditions can provide a more complete picture of the nature and magnitude of evolutionary adaptation following invasion.

Genome-wide SNPs used here provided much higher resolution than microsatellites in detecting population structure and dispersal history of this invasive species, as seen in other comparisons of these marker systems (Rasic et al. 2014; Fischer et al. 2017). The genetic structure of *T. palmi* populations in China follows geography with northern populations likely introduced from southern China, which is consistent with the historical records of northward dispersal and the microsatellite results for this species (Cao et al. 2019). In East Asia, native insects have usually taken hundreds of thousands of years to expand their range from source areas to their current distribution (Wei et al. 2015; Ye et al. 2020), while in contrast, some invasive species quickly spread across wide distribution areas within decades or even a few years (Biondi et al. 2018; Wu et al. 2018). *Thrips palmi* has taken about 2 decades to spread out from its introduction area in southern China to northern greenhouse environments, spreading rapidly compared to native species but not as quickly as some other invasive species (Cao et al. 2016).

The CT_max_ of open field–collected populations from southern China showed a decline with latitude but then a sharp increase following colonization of the greenhouse environment. The observed pattern of phenotype variation could be shaped by plasticity changes, genetic drift, and/or adaptive evolution. To distinguish adaptive evolution from acclimatization and other forms of plasticity, we here reared populations in the laboratory for 1 generation to reduce the influence of environmental variation. More generations might be required to overcome transgenerational effects, but these appear small for insect stress resistance compared to the effects of intragenerational plasticity (Sgro et al. 2016). A longer rearing period also introduces the risk of rapid adaptation to laboratory conditions often documented in insects (Hoffmann and Ross 2018). Genetic drift seems unlikely to explain our results because multiple populations were sampled and measured from each region, and population from the same region showed consistent trait patterns. The demographic analyses showed that all populations had similar patterns of historical *N*_e_, low LD, and limited gene flow after colonization, reducing the likelihood of genetic drift and human-mediated dispersal. Genetic changes generated by natural selection seem to contribute to the phenotypic patterns we observed. Selection experiment showed that thermal tolerance can be increased with only a few rounds of directional selection, supporting the potential for rapid evolution of CT_max_ over ecologically relevant timeframes. Greenhouses provide protection from low temperatures and seasonal adversity for growing crops, but the temperature inside greenhouses can be 20 to 30 °C higher than outside them (Shamshiri et al. 2018), which can lead to thermal stress for insects in greenhouses, consistent with higher levels of heat tolerance in these populations. Overall, the observed clinal variation of heat tolerance in *T. palmi* and differences between field and greenhouse environments are therefore most likely due to rapid and repeated adaptive evolution.

Rapid adaptive evolution has been increasingly reported in plants in their introduced range (Helliwell et al. 2018; van Boheemen et al. 2019; Sun et al. 2020; Battlay et al. 2023). Insects often have a shorter lifespan and more generations per year compared to plants, so perhaps genetic adaptation is even more likely than in plants under a short timeframe, particularly in insect species like thrips with low movement rates. It would therefore be interesting to explore the prevalence of adaptive evolution in a range of insect invaders with different life history traits across various environments (Pélissié et al. 2022; Sherpa et al. 2022; Dai et al. 2023).

Patterns of genetic diversity and demographic history across a species’ range are shaped from a complex interplay between the diversifying effect of demographic variation across landscapes with different selection pressures and the homogenizing effects of dispersal (Smith et al. 2020). Thrips are easily transferred across regions by human activities due to their small body size and cryptic habits, leading to many thrips becoming global invaders outside their native ranges (Morse and Hoddle 2006). Passive movement usually leads to a lack of geography-associated population genetic structure in the species since the transporting direction is not always restricted to nearby regions; this phenomenon was reported in the western flower thrips *Frankliniella occidentalis*, which took less than 10 yr to reach its maximum distribution range after its introduction to China (Wu et al. 2018). Population genetic analysis of *F. occidentalis* showed that geographically distant populations can share the same genetic cluster while closely related populations can come from different sources (Cao et al. 2017).

As a common pest of many vegetables, *T. palmi* is also very likely to be dispersed across distant regions by transportation of host plants, but we observed a stepping-stone dispersal in this species. While an initial response to a new environment might involve phenotypic plasticity rather than evolutionary processes, which tend to be slower (Hoffmann et al. 2005; Chevin and Lande 2010; Fox et al. 2019; Stamp and Hadfield 2020), evolutionary changes may be more likely in species with gradual range expansion rather than those dispersing rapidly and widely. Thus, evolution may play a particularly important role in stepping-stone dispersing species, with signatures of local adaptation often identified in such species (Dai et al. 2023). Successful establishment of transported individuals depends on preadaptation to the local environment if there is limited adaptive plasticity (Sgro et al. 2016; Gibert et al. 2019). When species are adapted to their native range, their ability to expand into new areas can then be hindered due to a mismatch between environments, particularly if gene flow is limited (Schlaepfer et al. 2002; Kellermann et al. 2009; Cao et al. 2021). The lack of recent gene flow among populations from different regions supports the notion that occasional long-distance movement does not have much impact on overall rates of gene flow (Cao et al. 2021).

Genetic diversity provides a source of variation for populations adapting to rapid environmental change (Lande and Shannon 1996; Thomas et al. 2017). Ancestral insect populations usually possess higher genetic diversity than derived populations (Wei et al. 2015). In *T. palmi*, high genetic diversity was found in 2 populations from Yunnan province, supporting the assumption that Yunnan is in the native range of this species in China. Derived populations of an invader with lower variation could have a more restricted adaptive capacity (Bock et al. 2015). Repeated introductions may help overcome this issue and facilitate adaptive responses to new environments (van Boheemen et al. 2017; Smith et al. 2020). In this sense, we note that the colonized northern populations of *T. palmi* have a higher level of genetic admixture than southern populations, which may point to repeated introduction from different sources that could impact local adaptation, such as seen when populations shifted from open field to greenhouse conditions.

Uncovering the genetics of climate adaptation is a major concern in ecological genetics (Franks and Hoffmann 2012; Bomblies and Peichel 2022). Here, we discovered 3 large chromosome inversions that exhibited varying levels of association with heat stress tolerance in field and laboratory-selected populations. A relationship between chromosome inversions and climate change has been previously noted in *Drosophila* (Wesley and Eanes 1994; Umina et al. 2005; Kapun and Flatt 2019), and a growing number of studies have shown that chromosome inversions have a significant impact on adaptation (Mérot et al. 2020). Moreover, inversions have been implicated in local climate adaptation following a recent invasion (Galludo et al. 2018). Recent advances in genome sequencing technology and analyses have made the identification of inversions feasible in nonmodel species (Chakraborty et al. 2018; Wellenreuther et al. 2019). However, challenges remain in identifying inversions. Due to the high cost of directly identifying inversions through long-read sequencing (De Coster et al. 2021; Bock et al. 2023), we here primarily utilized indirect methods (Cohen et al. 2023) such as local PCA and heterozygosity analyses, along with SNPs developed from short-read sequencing to detect chromosomal inversions. We conducted whole-genome resequencing at a high depth of approximately 45×, which improved the accurate detection of SNPs inside and outside the inversions. The identified inversions ranged in size from 667 to 1,130 kb; such large inversion can be relatively easily detected using an indirect method (Mérot et al. 2020).

Fisher's micromutational view predicts that adaptive traits usually have a polygenic architecture that involves many loci of small effects and few loci of large effects (Fisher 1930; Orr 2005; Connallon and Hodgins 2021). However, large effect loci could still be favored in populations under extreme environmental stress that deviate a population far from the optimum (Orr 1998; Bomblies and Peichel 2022). Under polygenic adaptation, large haplotype blocks can preserve clusters of coselected genes that segregate as single alleles of large effects (Kirkpatrick and Barton 2006; Kirkpatrick and Barrett 2015). Previous work provides compelling evidence that several inversions in *Drosophila melanogaster* and *D. subobscura* are adaptive (Galludo et al. 2018; Kapun and Flatt 2019). In invasive plants, large haplotype blocks contribute substantially to genetic signals of rapid and parallel adaptation (Kapun and Flatt 2019). We considered the inverted region as an individual locus and associated its frequency with CT_max_ values in geographical populations, which is a more powerful approach to finding associations with phenotypic and environmental variation (Mérot et al. 2020; Todesco et al. 2020). We observed a higher frequency of 2 inversions in the laboratory-selected population, which changed as CT_max_ increased, providing another source of evidence for inversion involvement. Given the challenge of determining whether sequence differentiation associated with chromosomal arrangements has adaptive value rather than the result of demography affecting population structure (Mérot et al. 2020), additional data would be useful, such as associating seasonal variation in chromosome inversion frequencies with phenotypes and climate variables in populations where chromosomal arrangements are polymorphic.

Our genome scans identified a large number of genes potentially affecting adaptation to thermal stress. Among analyses using different population groups and methods, outlier genes showed overlapping enrichment of functions, such as cytoskeletal organization, oxidation–reduction, and heat stress response. However, outlier genes were rarely shared between comparisons. Beyond potentially large effect alleles such as those influencing heat shock protein expression, evolutionary adaptation to extremely high temperature is a high-level trait that can be influenced by nearly all biochemical and biophysical aspects of the organism, leaving many low-level phenotypes and genotypes under selection in the evolutionary process (Somero 1995). The shared functions of outlier genes point to conserved pathways in heat stress adaptation, as identified in previous biochemical, biophysical, and evolutionary studies (Bomblies and Peichel 2022). The diverse genes identified in different population groups may indicate that selection occurred at different time points in these populations (Therkildsen et al. 2013), but even replicate populations with the same background exposed to the same directional climate-related selection pressures at the same time can produce selection responses involving different sets of alleles (Griffin et al. 2017).

In conclusion, we found that the invasion of an invasive pest over the last few decades follows a stepping-stone dispersal, unlike some other invasive pests where there is little association between geographic and genetic distance. This invasion process has been associated with evolutionary changes in thermal tolerance consistent with expected clinal variation in climate and the unusual thermal conditions imposed by a greenhouse habitat in northern areas. Chromosome inversions may play a role in rapid response to temperature changes, reiterating the importance of structural variation as large effect alleles in local adaptation. There was little consistency in outlier genes associated with climate adaptation in different comparisons but overlap with their functional effects, reflecting the complex processes of selection and genetic architecture involved in local adaptation.

## Materials and Methods

### Samples and Insect Rearing

We used 22 populations of *T. palmi* in our study, of which 19 were collected from China, 1 was collected from Japan, and 2 were selected through exposure to heat stress in the laboratory as described below (supplementary table S1, Supplementary Material online; Fig. 1). In northern China, populations were collected from greenhouses, where *T. palmi* could not overwinter in open fields, while in southern and central China, populations were collected from fields. In total, 16 populations collected from open fields and greenhouses of China, one collected from Japan, and two laboratory-selected populations were used for whole-genome resequencing. Because *T. palmi* has a haplodiploid sex determination system, in which haploid individuals develop into males while diploid individuals develop into females, diploid females were genotyped for estimating heterozygosity. Fifteen female adults from each population were used for genome resequencing; these had been collected from different plants with individuals separated by 5 m to reduce the chance of collecting siblings.

We used 11 populations collected from open fields and greenhouse (18.41°N to 40.71°N) and 2 laboratory-selected populations for phenotypic measurements. These field- and greenhouse-collected populations were reared in the laboratory at 25 °C, with a relative humidity of 50% ± 5% and a photoperiod of 16L:8D (Kawai 1990; Yadav and Chang 2014), for testing thermal tolerance in common garden experiments. Each laboratory population was established from more than 1,000 male and female adults (the sex ratio in the field is typically female biased with twice as many females as males). Eggs were laid on seedlings of cucumber while larvae were reared on pods of common bean, *Phaseolus vulgaris*, in a jar sealed by a 200-μm mesh net. Two-day-old female adults of the F_1_ generation were used in the stress tests.

### Genome Resequencing and SNP Calling

High-quality genomic DNA was extracted for each individual of *T. palmi* with a DNeasy Blood & Tissue Kit (Qiagen, Hilden, Germany), following the manufacturer’s instructions. Illumina sequencing libraries were constructed for each individual using the TruSeq Nano DNA Library Prep Kits and sequenced on an Illumina NovaSeq 6000 platform to generate 150 bp paired-end reads. The raw sequencing reads were filtered using FASTP version 1.2.1 (Chen et al. 2018). Paired-end clean reads were mapped to the *T. palmi* reference genome using BWA-MEM version 0.7.12 (Li and Durbin 2010; Guo et al. 2020). SAMtools version 1.16 was used to mark and remove duplicate reads (Li et al. 2009). SNP calling was performed using GATK version 4.0 (McKenna et al. 2010). We first conducted hard filtering in GATK to remove false variants with parameters as follows: QD < 2.0 || MQ < 40.0 || FS > 60.0 || SOR > 3.0 || MQRankSum < −12.5 || ReadPosRankSum < −8.0. Then, the R packages *vcfR* and VCFtools version 0.1.16 (Danecek et al. 2011) were used to exclude SNPs with nonbiallelic allele, minor allele frequency < 0.05, and missing rate > 20% and and to exclude individuals with SNP missing rate > 20%. The final set of SNPs was functionally annotated by SnpEff version 4.3 (Cingolani et al. 2012).

### Population Genetic Diversity and Demographic Analysis

Nucleotide diversity (*π*) was unbiasedly calculated by pixy v1.0.4. (Korunes and Samuk 2021) based on an all-site data set including variant sites, invariant sites, and missing sites. Proportion of polymorphic loci, observed heterozygosity (*H*_o_), expected heterozygosity (*H*_e_), and inbreeding coefficient (*F*_IS_) were estimated using the program “Populations” implemented in STACKS v2 (Catchen et al. 2013), following the data set preparation method of Schmidt et al. (2021), which considers monomorphic as well as polymorphic nucleotides across autosomes for each population with all missing sites removed.

SMC++ version 1.15.2 was used to infer the historical variation of the effective population size (Terhorst et al. 2017). We assumed a mutation rate of 2.9 × 10^−9^ per site per generation as estimated for *Heliconius* (Lepidoptera: Hesperiidae; Keightley et al. 2015) and a generation time of 0.06 yr. Pairwise LD of genome-wide SNPs was calculated for each population based on allele frequency correlations (*r*^2^) using PopLDdecay version 3.42 (Zhang et al. 2019).

PCA and an admixture model with correlated allele frequencies implemented in ADMIXTURE v.1.3.0 (Alexander et al. 2009) were used to infer the population genetic structure. The optimal number of ancestral lineages (*K*) was set from 1 to 10 in the ADMIXTURE analysis.

Demographic history among populations was inferred using KimTree version 1.3 (Gautier and Vitalis 2013). To reduce the complexity of the tested scenarios, a hierarchical approach by optimizing the procedure in a series of steps was applied. First, relationships among the 4 main clusters of ADMIXTURE analysis were constructed based on 4 representative populations. Then, the other genetic clusters divided from the 4 main clusters were individually added to the optimal tree topology. Support for the different scenarios was assessed using a deviance information criterion (DIC; Gautier and Vitalis 2013). To examine whether there is isolation by distance (IBD), we conducted a Mantel test to correlate the linearized pairwise genetic differentiation of all variations [i.e. *F*_ST_/(1 − *F*_ST_)] with geographical distance and climatic dissimilarity using the R package *ade4* version 1.7-15, with 10,000 permutations. For climatic dissimilarity, bioclimatic variables (bio1 to bio19) related to temperature and precipitation at a resolution of 5-degree minutes were retrieved from the WorldClim database (https://worldclim.org/). Then, we inferred population split among Chinese populations by considering historical gene flow using TreeMix version 1.13 (Pickrell and Pritchard 2012) with 1 to 10 migration events. Based on the KimTree results, 2 populations from Yunnan were used as outgroups. To reduce the effects of linkage on population genetic structure inferences, we used SNPs scattered in the genome with a distance >20,000 bp for analysis.

### Testing Geographic and Climatic Effects on Population Differentiation

RDA was conducted to quantify the specific and combined effects of landscape elements on adaptive divergence, namely partition genomic variation into components explained by genetic structure, geographic distance, climate variables, and their combination. For genetic structure, neutral variants were used to create a PCA object to get the number of proxies of neutral genetic structure. The bioclimatic variables with a variance inflation factor (VIF) higher than 10 were excluded to avoid multicollinearity. Both full (genetic structure, climate, and geography) and partial (genetic structure, climate, or geography) models of RDA were analyzed using the function *rda* in the R package *vegan*. The independent effect of the genetic structure, climate, or geography was the variance values for the constrained matrix of the other 2 groups of variables in the partial model. The collinear proportion was calculated by subtracting the independent effects of genetic structure, climate, and geography from the total amount of variance explained in the full RDA model.

### Heat and Cold Tolerance Tests

The heat tolerance of *T. palmi* was measured using CT_max_, while the cold tolerance was measured by CCRT. For the CT_max_ test, adults of *T. palmi* were allowed to settle down at 25 °C for 5 min (same as temperature in the common garden experiment), and then the temperature was gradually increased by 0.2 °C/min until *T. palmi* were knocked down. CT_max_ was recorded as the mean knockdown temperature for each population at this ramping rate. CT_max_ of at least 100 individuals was measured for each geographical population. For the CCRT test, 35 adults were moved into a plastic cup and then knocked down at a constant temperature of −7 °C for 1 h in a temperature test chamber (GD(J)S-100, Beijing Yashilin Testing Equipment Co., Ltd., Beijing, China). The time to recovery of the treated individuals was visually scored at 25 °C. Thrips were considered recovered when the treated thrips moved normally for a distance of >3× their body length. Three replicates were conducted for each population.

### Laboratory Selection of a Field Population under High-Temperature Stress

About 5,000 *T. palmi* adults of the BJDX population that had been reared for a generation in the laboratory were placed under a heat stress that killed about 50% of individuals. All thrips were put into a plastic box and then moved to the temperature test device (GD(J)S-100, Beijing Yashilin Testing Equipment Co., Ltd., Beijing, China). After keeping the thrips at 25 °C for 5 min, the temperature was gradually raised at a rate of 0.2 °C/min, and the procedure was stopped at 88 min. Three thousand adults of the next 3 generations were exposed to selection in the same way by ramping the temperature for increasing period up to 98 min to achieve 50% mortality. The CT_max_ and CCRT before and after selection were measured using methods described above.

The realized heritability (*h*^2^) was estimated as described in Tabashnik (1992):


(1) $$h^{2}=R/S.$$

*R* denotes selection response and was estimated as follows:


(2) $$R=\frac{\mathrm{Log}\;CT_{\max}\;(F4)-\mathrm{Log}\;CT_{\max}\;(F1)}{n},$$

where *n* shows generations screened with high temperatures.

*S* denotes selection differential and was calculated as follows:


(3) $$S=i\times \sigma_{p},$$

where *i* denotes selection intensity estimated from the percent survival of selection (*p*) using the selection intensity table reported by Falconer (1989). *σ_p_* denotes phenotypic deviation and is determined as follows:


(4) $$\sigma_{p}=\frac{1}{\mathrm{mean}\;\mathrm{slope}\;(F1-F4)}.$$

### GF Analysis

The GF method was used to describe the response of genetic variation to the gradient of climate variables (bio5, bio8, and bio10) and CT_max_. A data set composed of all SNPs potentially under selection was prepared by combing all SNPs identified in any of the outlier scanning methods as described below. The R package *gradientForest* (Breiman 2001; Ellis et al. 2012) was used to perform the GF analysis with 10,000 regression trees and a variable correlation threshold of 0.5. The turnover functions were conducted by GF analysis to describe the response of genetic variation to the gradient of climate variables (Fitzpatrick and Keller 2015).

### Chromosome Inversion Analysis

Structural genomic variations across populations were scanned using a local PCA and LD analysis, following Todesco et al. (2020).The R package *lostruct* v.0.0.0.9 (Li and Ralph 2018) was used to compute the local PCA coordinates of each nonoverlapping 100 SNP-wide window for each chromosome. Then, the distance matrix between windows was calculated and visualized by plotting the multidimensional scaling (MDS) transformation score against each window in the chromosome. The recombination suppression regions were manually selected based on minimum or maximum MDS values and validated by PCA, heterozygosity, and LD analysis using SNPs within the potential regions. The PCA and heterozygosity of the potential inverted regions were expected to divide samples into 3 groups representing 0/0, 0/1, and 1/1 genotypes. The program vcftools v0.1.3 was used to calculate the squared correlation coefficient (*r*^2^) between genotypes, which was transformed to mean values for each 50-kb window.

### Population Differentiation Analysis

Two population differentiation methods were used to identify outliers. First, a weighted kNN method (Pfeifer et al. 2020) was used to scan outliers putatively under selection in all 14 populations, the field and greenhouse groups. This method uses pairwise *F*_ST_ values from all population comparisons to define the locations of all loci in multidimensional space (Pfeifer et al. 2020). SNPs putatively under selection were identified within the 0.01 quantile. Second, outliers were scanned by pairwise genetic differentiation (*F*_ST_). Two methods were used to acquire the *F*_ST_ values. In one of these, *F*_ST_ values were calculated by pixy v1.0.4.beta1 (Korunes and Samuk 2021) over 2-kb nonoverlapping windows based on all invariant sites and variant sites. In the other approach, VCFtools version 0.1.16 (Danecek et al. 2011) was used to calculate *F*_ST_ values from sample allele frequency likelihoods over 2-kb sliding windows with overlapping 2 kb across the genome according to the results of the LD decay estimation (supplementary fig. S3C and D, Supplementary Material online). For both methods, sliding windows with the top 0.2% of *F*_ST_ values were selected as significant windows and further scanned for outliers.

### Association Analysis

A univariate LFMM implemented in the R package LEA version 2.8.0 (Frichot and François 2015) was used to examine associations between allele frequencies and mean CT_max_ of each group. LFMM analyses were performed with 4 latent factors (*K* = 4) to account for population structure in the genotype data based on the number of ancestry clusters inferred with admixture version 1.3.0 (Alexander et al. 2009). The LFMM analysis was conducted for each predictor with 4 latent factors, 10,000 iterations, 5,000 burn-ins, and 4 replicates. SNPs with a threshold of 0.000001 and 0.00001 were chosen as candidate outliers for the field and greenhouse groups, respectively. Functions of outlier genes were extracted from the genome annotation (Guo et al. 2020).

## Supplementary Material

Supplementary material is available at *Molecular Biology and Evolution* online.

## Supplementary Material

msae044_Supplementary_Data

## Data Availability

The genome resequencing project for *T. palmi* was deposited at NCBI under BioProject No. PRJNA1025253. The Illumina short-read sequences generated in this study were deposited in Sequence Read Archive (SRA) of GenBank under accession numbers SAMN37809732 to SAMN37809980. The scripts and commands used in this study have been deposited in the GitHub repository (https://github.com/gentlewasp/population_genomics_climate_adaptation_scripts). The input files for data analyses have been deposited in the Figshare repository (https://doi.org/10.6084/m9.figshare.25047020).
