# Supplementary material for: Rapid and Repeated Climate Adaptation Involving Chromosome Inversions following Invasion of an Insect
Source: Mol Biol Evol. 2024 Feb 24;41(3):msae044. doi: 10.1093/molbev/msae044 (PMC10924284; doi:10.1093/molbev/msae044)
Supplement: msae044_Supplementary_Data [file msae044_supplementary_data.zip › LijunMa-supplementaryInfor-r1.docx]

**Supplementary Information**

**Rapid and repeated climate adaptation involving chromosome inversions following invasion of an insect**

Li-Jun Ma ^1^, Li-Jun Cao ^1^, Jin-Cui Chen ^1^, Meng-Qing Tang ^1,2^, Wei Song ^1^, Fang-Yuan Yang ^1^, Xiu-Jing Shen ^1^, Ya-Jing Ren ^1,2^, Qiong Yang ^3^, Hu Li^2^, Ary Anthony Hoffmann ^3, *^, Shu-Jun Wei ^1,^ *

1. Institute of Plant Protection, Beijing Academy of Agriculture and Forestry Sciences, Beijing 100097, China

2. Department of Entomology and MOA Key Lab of Pest Monitoring and Green Management, College of Plant Protection, China Agricultural University, Beijing 100193, China

3. Bio21 Institute, School of BioSciences, University of Melbourne, Parkville, Victoria 3010, Australia

*Correspondence authors:

Shu-Jun Wei

Institute of Plant Protection, Beijing Academy of Agriculture and Forestry Sciences, 9 Shuguanghuayuan Middle Road, Haidian District, Beijing 100097, China. Tel: +86 010-51503439; E-mail: shujun268@163.com

Ary Anthony Hoffmann

Bio21 Institute, School of BioSciences, University of Melbourne, Parkville, Victoria 3010, Australia; E-mail: ary@unimelb.edu.au

**Table S1** Sampling information and data collected for the 22 populations of *T. palmi*.

| **Population code** | **Collection**  **location** | **CT_max_ (minute)** | **Genomic data** | **Longitude (E)** | **Latitude (N)** | **Altitude**  **(m)** | **Sampling**  **date** | **Host**  **plant** |
| --- | --- | --- | --- | --- | --- | --- | --- | --- |
| JANP/JAPN | Okinawa-ken, Japan | - | Yes | 127.8333 | 26.3333 | 45 | 2016/01/01 | Cucumber |
| LNAS | Anshan, Liaoning | - | Yes | 123.0078 | 41.1187 | 57 | 2017/09/27 | Eggplant |
| NMHS | Hohhot, Inner Mongolia | 94.72 | Yes | 111.7992 | 40.7131 | 1058 | 2020/08/31 | Eggplant |
| BJCY | Chaoyang, Beijing | - | Yes | 116.5217 | 39.9590 | 36 | 2017/10/27 | Eggplant |
| BJDX | Daxing, Beijing | 99.14 | Yes | 116.3109 | 39.6146 | 35 | 2020/10/21 | Cucumber |
| BJFS | Fangshan, Beijing | 98.99 | No | 115.9576 | 39.6117 | 39 | 2020/9/22 | Cucumber |
| SJY1 | Daxing, Beijing | 99.14 | Yes | 116.3109 | 39.6146 | 35 | 2020/10/21 | Cucumber |
| SJY4 | Daxing, Beijing | 108.13 | Yes | 116.3109 | 39.6146 | 35 | 2020/10/21 | Cucumber |
| SDSG | Shouguang, Shandong | - | Yes | 118.9578 | 36.8642 | 10 | 2018/11/13 | Cucumber |
| SDS3/SDWF | Shouguang, Shandong | 110.02 | Yes | 118.7000 | 36.8036 | 32 | 2020/12/21 | Cucumber |
| SDSG2/SDS2 | Shouguang, Shandong | 107.11 | No | 118.8965 | 37.2439 | -11 | 2020/12/22 | Cucumber |
| SDS1/SDST | Shouguang, Shandong | 107.63 | Yes | 118.7608 | 36.7862 | 29 | 2020/10/13 | Cucumber |
| SCCD | Chengdu, Sichuan | - | Yes | 103.5427 | 31.2427 | 2213 | 2018/08/21 | Eggplant |
| SCDY | Deyang, Sichuan | 97.11 | Yes | 104.4264 | 30.9771 | 476 | 2020/09/02 | Eggplant |
| HNCS | Changsha, Hunan | - | Yes | 113.1957 | 28.2380 | 66 | 2017/07/21 | Eggplant |
| GDZQ | Zhaoqing, Guangdong | 104.48 | Yes | 112.6669 | 23.0525 | 32 | 2020/10/28 | Cucumber |
| GDSZ | Shenzhen, Guangdong | - | Yes | 114.3477 | 22.6575 | 173 | 2018/04/21 | Eggplant |
| YNBN | Xishuangbanna, Yunnan | 102.11 | Yes | 100.2449 | 21.9430 | 1210 | 2020/09/19 | Watermelon |
| YNXS | Xishuangbanna, Yunnan | - | Yes | 100.7512 | 21.7048 | 602 | 2018/04/15 | Eggplant |
| HNSY | Sanya, Hainan | - | Yes | 109.1875 | 18.4304 | 93 | 2018/03/08 | Eggplant |
| HNS1/HNSN | Sanya, Hainan | 105.25 | Yes | 109.1981 | 18.4088 | 26 | 2020/12/30 | Eggplant |
| HNSY2/HNS2 | Sanya, Hainan | 116.07 | No | 109.2112 | 18.3439 | 99 | 2020/12/30 | Luffa |

Note: BJDX is the code for the field-collected population used in selection; CT_max_ (drop time in minutes) was tested on the F1 generation, coded as SJY1, and the population was selected three times for heat stress in the laboratory (SJY4).

**Table S2** Summary of generated sequencing data for the 249 samples of *Thrips palmi.*

| **Sample** | **Clean reads** | **Mapping ratio** | **Depth (X)** | **Properly paired ratio** |
| --- | --- | --- | --- | --- |
| BJCY01 | 97016372 | 85.85% | 53.97 | 78.32% |
| BJCY02 | 82833962 | 79.83% | 42.72 | 72.99% |
| BJCY03 | 106894482 | 82.34% | 56.92 | 72.36% |
| BJCY04 | 93545146 | 85.63% | 51.91 | 78.19% |
| BJCY06 | 93524032 | 90.07% | 54.65 | 82.29% |
| BJCY07 | 86082970 | 91.38% | 50.94 | 81.79% |
| BJCY08 | 75027440 | 87.47% | 42.45 | 77.59% |
| BJCY09 | 76982000 | 91.31% | 45.60 | 83.82% |
| BJCY10 | 81743458 | 90.41% | 47.95 | 83.25% |
| BJCY11 | 95397164 | 88.69% | 54.86 | 80.98% |
| BJCY12 | 71615944 | 90.55% | 42.08 | 82.83% |
| BJCY13 | 92773632 | 91.57% | 55.19 | 83.24% |
| BJCY14 | 79838690 | 89.53% | 46.33 | 78.88% |
| BJCY15 | 82388272 | 90.90% | 48.55 | 83.68% |
| BJCY16 | 86951794 | 91.18% | 51.49 | 85.36% |
| BJDX01 | 49467196 | 90.82% | 29.16 | 84.77% |
| BJDX02 | 77241566 | 91.28% | 45.82 | 83.49% |
| BJDX03 | 77764334 | 81.06% | 40.72 | 74.58% |
| BJDX04 | 87805696 | 87.85% | 50.00 | 81.14% |
| BJDX05 | 76327820 | 92.13% | 45.68 | 85.22% |
| BJDX06 | 69977266 | 83.97% | 38.02 | 76.34% |
| BJDX07 | 54388924 | 79.31% | 27.90 | 72.37% |
| BJDX08 | 62569824 | 83.74% | 33.92 | 74.44% |
| BJDX09 | 78642450 | 71.82% | 36.39 | 66.18% |
| BJDX10 | 74087176 | 93.29% | 44.92 | 84.36% |
| BJDX11 | 79290472 | 65.16% | 33.21 | 57.50% |
| BJDX12 | 71115550 | 89.42% | 41.29 | 79.50% |
| BJDX13 | 63694308 | 87.77% | 36.25 | 80.26% |
| BJDX14 | 69454694 | 89.75% | 40.46 | 81.43% |
| BJDX15 | 88946872 | 93.20% | 53.81 | 85.62% |
| GDSZ01 | 115472396 | 93.05% | 70.11 | 84.21% |
| GDSZ02 | 125723254 | 91.27% | 74.82 | 81.58% |
| GDSZ03 | 121149706 | 92.93% | 73.41 | 85.38% |
| GDSZ04 | 117196676 | 93.28% | 71.25 | 84.95% |
| GDSZ05 | 93368840 | 87.52% | 53.14 | 81.12% |
| GDSZ06 | 87723660 | 93.21% | 53.55 | 86.64% |
| GDSZ07 | 121589088 | 93.35% | 74.12 | 82.43% |
| GDSZ08 | 83667532 | 93.28% | 50.94 | 86.16% |
| GDSZ09 | 52072088 | 89.22% | 30.30 | 79.96% |
| GDSZ10 | 83502092 | 92.56% | 50.42 | 86.78% |
| GDSZ11 | 98553588 | 87.48% | 56.16 | 80.53% |
| GDSZ12 | 94418940 | 91.04% | 56.00 | 82.27% |
| GDSZ13 | 73432444 | 90.04% | 43.04 | 82.97% |
| GDSZ14 | 89512104 | 92.63% | 54.05 | 84.95% |
| GDSZ15 | 92740436 | 91.14% | 55.06 | 83.75% |
| GDZQ02 | 84833642 | 90.35% | 49.90 | 83.46% |
| GDZQ05 | 79195638 | 89.62% | 46.16 | 82.56% |
| GDZQ07 | 68245222 | 92.17% | 40.95 | 84.80% |
| GDZQ08 | 61029080 | 90.95% | 36.10 | 84.13% |
| GDZQ10 | 61952414 | 91.00% | 36.82 | 83.86% |
| GDZQ11 | 72985900 | 92.31% | 43.90 | 84.70% |
| GDZQ12 | 90674108 | 89.44% | 52.69 | 82.49% |
| GDZQ13 | 64944428 | 91.95% | 38.84 | 85.63% |
| GDZQ14 | 70964642 | 92.00% | 42.53 | 84.62% |
| GDZQ15 | 59953674 | 77.14% | 29.91 | 71.18% |
| HNCS01 | 95737674 | 92.89% | 57.92 | 84.86% |
| HNCS02 | 85965392 | 84.54% | 47.11 | 78.19% |
| HNCS03 | 95054984 | 88.09% | 54.37 | 82.22% |
| HNCS04 | 74985532 | 93.00% | 45.37 | 86.69% |
| HNCS05 | 93050768 | 93.35% | 56.53 | 85.46% |
| HNCS06 | 94640752 | 93.46% | 57.68 | 86.53% |
| HNCS07 | 113551254 | 89.07% | 65.72 | 81.12% |
| HNCS08 | 78395116 | 93.43% | 47.71 | 84.84% |
| HNCS09 | 84642496 | 94.40% | 51.99 | 87.83% |
| HNCS10 | 119301956 | 92.53% | 71.91 | 86.25% |
| HNCS11 | 83948034 | 93.33% | 50.97 | 86.15% |
| HNCS12 | 99852524 | 92.84% | 60.38 | 84.97% |
| HNCS13 | 138216448 | 93.35% | 84.04 | 84.98% |
| HNCS14 | 108968996 | 93.12% | 66.09 | 85.09% |
| HNCS15 | 92725630 | 87.95% | 52.97 | 81.22% |
| HNS101 | 54147974 | 94.31% | 33.35 | 87.10% |
| HNS102 | 66669552 | 93.74% | 40.81 | 86.12% |
| HNS103 | 68748580 | 91.58% | 41.10 | 83.09% |
| HNS104 | 43605824 | 94.10% | 26.79 | 87.33% |
| HNS105 | 70035202 | 66.25% | 29.87 | 60.75% |
| HNS106 | 67839840 | 92.74% | 41.09 | 83.54% |
| HNS107 | 83414236 | 91.96% | 50.06 | 83.56% |
| HNS108 | 88192686 | 88.41% | 50.82 | 79.63% |
| HNS109 | 82595374 | 92.57% | 49.97 | 82.04% |
| HNS110 | 78992954 | 87.43% | 45.02 | 78.86% |
| HNS111 | 82767538 | 92.07% | 49.68 | 83.64% |
| HNS112 | 83968318 | 52.60% | 28.30 | 47.70% |
| HNS113 | 67441332 | 91.88% | 40.41 | 84.30% |
| HNS114 | 60616756 | 93.89% | 37.19 | 85.82% |
| HNS115 | 34193276 | 93.51% | 20.90 | 86.47% |
| HNSY01 | 86377234 | 52.12% | 28.90 | 46.85% |
| HNSY03 | 114904504 | 41.91% | 30.80 | 37.53% |
| HNSY04 | 84957208 | 54.73% | 29.89 | 49.11% |
| HNSY05 | 115622464 | 56.27% | 41.84 | 50.53% |
| HNSY08 | 106595458 | 47.28% | 32.29 | 42.71% |
| HNSY09 | 104142084 | 72.30% | 48.75 | 64.26% |
| HNSY10 | 95781314 | 64.87% | 40.09 | 58.45% |
| HNSY12 | 93644570 | 65.30% | 39.47 | 58.63% |
| HNSY13 | 80752856 | 80.86% | 42.43 | 73.00% |
| HNSY14 | 93040094 | 65.81% | 39.53 | 59.26% |
| HNSY15 | 80219086 | 75.43% | 39.20 | 67.72% |
| JANP01 | 87839630 | 84.39% | 48.24 | 77.28% |
| JANP03 | 91801546 | 80.28% | 47.78 | 76.08% |
| JANP04 | 109848840 | 90.29% | 64.73 | 83.51% |
| JANP05 | 67878168 | 82.16% | 36.23 | 75.77% |
| JANP06 | 95377904 | 74.93% | 46.27 | 69.41% |
| JANP07 | 81120322 | 64.96% | 33.97 | 59.70% |
| JANP08 | 87485708 | 84.24% | 47.94 | 77.37% |
| JANP09 | 86207774 | 88.98% | 50.04 | 81.94% |
| JANP11 | 68911546 | 87.34% | 39.19 | 80.60% |
| JANP12 | 73622500 | 73.31% | 34.93 | 67.61% |
| JANP13 | 82584030 | 78.55% | 42.07 | 71.89% |
| JANP14 | 84266662 | 70.78% | 38.53 | 65.51% |
| JANP15 | 75381252 | 85.75% | 42.06 | 78.85% |
| LNAS01 | 71376446 | 90.76% | 42.04 | 82.55% |
| LNAS02 | 79268930 | 92.34% | 47.51 | 84.32% |
| LNAS03 | 65096444 | 92.93% | 39.28 | 84.84% |
| LNAS04 | 73924996 | 92.70% | 44.45 | 84.74% |
| LNAS05 | 82932592 | 93.66% | 50.44 | 85.59% |
| LNAS06 | 89314928 | 84.04% | 48.60 | 76.89% |
| LNAS07 | 83363630 | 92.80% | 50.25 | 84.45% |
| LNAS08 | 89687572 | 93.89% | 54.67 | 86.21% |
| LNAS09 | 100051702 | 94.09% | 61.11 | 86.62% |
| LNAS10 | 75577328 | 81.20% | 39.65 | 74.29% |
| LNAS11 | 80592970 | 82.46% | 42.94 | 76.59% |
| LNAS12 | 81021608 | 85.83% | 45.00 | 79.80% |
| LNAS13 | 88294542 | 86.82% | 49.71 | 81.12% |
| LNAS14 | 82743844 | 66.58% | 35.41 | 62.21% |
| LNAS15 | 91026902 | 72.13% | 42.28 | 66.97% |
| NMHS01 | 68344590 | 71.62% | 31.54 | 65.27% |
| NMHS02 | 50116530 | 87.70% | 28.51 | 79.98% |
| NMHS03 | 88415714 | 75.12% | 42.90 | 67.98% |
| NMHS04 | 100478900 | 89.02% | 58.10 | 80.57% |
| NMHS05 | 65107500 | 85.87% | 36.21 | 79.01% |
| NMHS06 | 84496806 | 87.50% | 47.93 | 80.26% |
| NMHS07 | 86184748 | 76.28% | 42.46 | 69.64% |
| NMHS08 | 82164600 | 87.05% | 46.41 | 79.99% |
| NMHS09 | 69945100 | 92.06% | 41.87 | 85.07% |
| NMHS10 | 54256854 | 89.46% | 31.52 | 83.10% |
| NMHS11 | 65498464 | 84.01% | 35.60 | 78.19% |
| NMHS12 | 61125690 | 86.35% | 34.19 | 80.37% |
| NMHS14 | 61471884 | 70.82% | 28.03 | 65.83% |
| SCCD01 | 119379966 | 92.38% | 71.58 | 82.65% |
| SCCD02 | 125142658 | 92.09% | 74.86 | 81.85% |
| SCCD03 | 137997904 | 93.34% | 83.79 | 83.54% |
| SCCD04 | 106299362 | 93.10% | 64.22 | 82.30% |
| SCCD05 | 100020340 | 93.37% | 60.63 | 82.25% |
| SCCD06 | 118996640 | 93.50% | 72.36 | 81.49% |
| SCCD07 | 101882644 | 81.68% | 53.89 | 71.99% |
| SCCD08 | 101760728 | 92.96% | 61.49 | 79.51% |
| SCCD09 | 87084926 | 93.52% | 52.99 | 85.26% |
| SCCD10 | 75961394 | 93.52% | 46.15 | 86.24% |
| SCCD11 | 97310884 | 91.50% | 57.91 | 82.41% |
| SCCD12 | 85403548 | 92.82% | 51.58 | 83.10% |
| SCCD13 | 99776712 | 93.16% | 60.38 | 85.08% |
| SCCD14 | 114645460 | 93.11% | 69.35 | 84.31% |
| SCCD15 | 69288494 | 94.05% | 42.41 | 85.69% |
| SCDY01 | 70853008 | 91.01% | 42.06 | 81.62% |
| SCDY02 | 73212818 | 83.80% | 39.85 | 76.41% |
| SCDY03 | 54113796 | 85.08% | 29.92 | 78.53% |
| SCDY04 | 66905048 | 91.16% | 39.77 | 83.98% |
| SCDY05 | 49544896 | 91.28% | 29.53 | 84.35% |
| SCDY06 | 49887616 | 91.98% | 29.90 | 84.79% |
| SCDY09 | 50473514 | 88.16% | 28.92 | 81.79% |
| SCDY11 | 68306960 | 78.84% | 34.92 | 70.89% |
| SCDY12 | 65352356 | 85.47% | 36.32 | 76.18% |
| SCDY13 | 69227990 | 85.84% | 38.65 | 77.92% |
| SCDY14 | 66709520 | 93.00% | 40.53 | 82.67% |
| SCDY15 | 65634464 | 89.76% | 38.40 | 81.55% |
| SDS101 | 57267580 | 88.55% | 32.92 | 81.52% |
| SDS102 | 68092488 | 89.41% | 39.52 | 81.18% |
| SDS106 | 48120050 | 93.87% | 29.36 | 87.89% |
| SDS107 | 47663848 | 93.61% | 29.02 | 87.10% |
| SDS108 | 41945808 | 93.41% | 25.45 | 87.51% |
| SDS110 | 47409350 | 85.90% | 26.38 | 80.52% |
| SDS111 | 45856334 | 89.01% | 26.47 | 82.65% |
| SDS113 | 51976086 | 93.25% | 31.47 | 87.21% |
| SDS114 | 55885552 | 93.46% | 34.00 | 86.50% |
| SDS115 | 44369424 | 89.83% | 25.86 | 84.09% |
| SDS307 | 63371092 | 88.56% | 36.42 | 81.40% |
| SDS308 | 82886064 | 79.61% | 42.65 | 72.99% |
| SDS309 | 73572070 | 85.11% | 40.53 | 78.28% |
| SDS310 | 70998290 | 85.30% | 39.25 | 78.39% |
| SDS311 | 77698086 | 83.82% | 42.17 | 76.90% |
| SDS312 | 86339052 | 86.99% | 48.65 | 80.39% |
| SDS314 | 87596316 | 88.04% | 49.98 | 81.25% |
| SDS315 | 59778762 | 87.02% | 33.73 | 80.45% |
| SDSG02 | 69897490 | 91.10% | 41.15 | 88.58% |
| SDSG03 | 74054570 | 84.84% | 40.47 | 82.62% |
| SDSG04 | 71281708 | 92.84% | 42.78 | 90.80% |
| SDSG05 | 59678482 | 92.61% | 35.76 | 90.51% |
| SDSG06 | 72159410 | 93.19% | 43.70 | 84.51% |
| SDSG07 | 77795772 | 92.45% | 46.80 | 83.14% |
| SDSG08 | 85311398 | 91.09% | 50.44 | 82.64% |
| SDSG09 | 50520112 | 93.01% | 30.52 | 84.77% |
| SDSG10 | 71583062 | 93.12% | 43.33 | 84.58% |
| SDSG11 | 80313830 | 65.06% | 33.59 | 59.34% |
| SDSG12 | 90827842 | 79.55% | 46.70 | 73.23% |
| SDSG14 | 82997438 | 83.29% | 44.76 | 77.67% |
| SDSG15 | 93025800 | 81.80% | 49.21 | 75.01% |
| SJY101 | 117637560 | 86.08% | 65.65 | 79.00% |
| SJY102 | 115724656 | 63.33% | 47.08 | 58.07% |
| SJY103 | 93516126 | 81.68% | 49.40 | 75.54% |
| SJY104 | 79108556 | 87.24% | 44.72 | 80.34% |
| SJY105 | 83792110 | 83.40% | 45.23 | 76.74% |
| SJY106 | 72127184 | 84.64% | 39.51 | 77.97% |
| SJY107 | 89815572 | 85.16% | 49.53 | 78.27% |
| SJY108 | 84338358 | 78.85% | 42.93 | 72.90% |
| SJY109 | 86200902 | 87.20% | 48.76 | 80.07% |
| SJY110 | 71643892 | 83.84% | 38.84 | 77.50% |
| SJY111 | 95268812 | 85.14% | 52.52 | 78.57% |
| SJY112 | 81892920 | 83.73% | 44.35 | 77.17% |
| SJY113 | 71038558 | 84.78% | 38.99 | 78.08% |
| SJY114 | 86654442 | 74.47% | 41.62 | 68.47% |
| SJY115 | 83142848 | 87.45% | 47.12 | 80.83% |
| SJY401 | 121756402 | 91.11% | 72.07 | 84.01% |
| SJY402 | 114680434 | 83.95% | 62.35 | 77.46% |
| SJY403 | 106833140 | 86.16% | 59.65 | 79.26% |
| SJY405 | 90363528 | 84.65% | 49.65 | 77.15% |
| SJY407 | 119882568 | 90.07% | 70.11 | 83.11% |
| SJY408 | 128972450 | 86.07% | 71.90 | 79.67% |
| SJY409 | 78577324 | 87.78% | 44.72 | 80.74% |
| SJY410 | 98275170 | 79.02% | 50.19 | 72.43% |
| SJY412 | 68477410 | 84.68% | 37.51 | 78.82% |
| SJY413 | 79422604 | 73.68% | 37.71 | 67.80% |
| SJY414 | 92870566 | 81.82% | 49.17 | 75.42% |
| YNBN02 | 52616012 | 89.86% | 30.92 | 82.63% |
| YNBN04 | 66390382 | 92.80% | 40.39 | 84.88% |
| YNBN05 | 79432760 | 91.91% | 47.85 | 82.88% |
| YNBN06 | 70501134 | 87.89% | 40.47 | 79.35% |
| YNBN07 | 79500826 | 91.90% | 47.88 | 82.11% |
| YNBN08 | 86720100 | 92.03% | 52.34 | 79.82% |
| YNBN09 | 79796842 | 86.53% | 45.11 | 77.32% |
| YNBN10 | 69971562 | 89.55% | 40.79 | 81.02% |
| YNBN11 | 87233988 | 92.32% | 52.71 | 79.33% |
| YNBN12 | 79924740 | 91.57% | 47.89 | 83.13% |
| YNBN13 | 79360278 | 94.03% | 48.92 | 86.60% |
| YNBN14 | 48063154 | 93.97% | 29.63 | 86.13% |
| YNBN15 | 75895386 | 93.30% | 46.41 | 85.31% |
| YNXS01 | 95960434 | 79.12% | 49.09 | 73.29% |
| YNXS02 | 104557754 | 67.09% | 45.33 | 60.77% |
| YNXS03 | 62938304 | 79.96% | 32.72 | 73.02% |
| YNXS04 | 102973948 | 77.20% | 51.60 | 70.45% |
| YNXS05 | 94908902 | 71.72% | 44.07 | 64.42% |
| YNXS06 | 98184254 | 70.52% | 44.87 | 63.86% |
| YNXS07 | 86963058 | 68.65% | 38.63 | 62.67% |
| YNXS08 | 92215800 | 71.24% | 42.43 | 65.92% |
| YNXS09 | 83695434 | 66.72% | 36.06 | 61.27% |
| YNXS10 | 94699502 | 83.13% | 51.26 | 75.53% |
| YNXS11 | 66603424 | 78.12% | 33.68 | 72.43% |
| YNXS12 | 88423874 | 79.97% | 45.98 | 72.78% |
| YNXS13 | 103262954 | 66.80% | 44.50 | 61.15% |
| YNXS14 | 75802550 | 66.54% | 32.54 | 61.34% |
| YNXS15 | 64980134 | 69.01% | 29.03 | 62.41% |

**Table S3** Genetic diversity measures and inbreeding levels of 19 populations of *Thrips palmi* based on SNPs and invariant sites.

| Population code | Diversity level | *π* | *H*_o_ | *H*_e_ | *F*_IS_ |
| --- | --- | --- | --- | --- | --- |
| JAPN | Very low | 0.0029 | 0.0468 | 0.03803 | -0.01499 |
| LNAS | Low | 0.0045 | 0.08055 | 0.07353 | -0.00823 |
| NMHS | Low | 0.0049 | 0.08647 | 0.07542 | -0.01606 |
| BJCY | Low | 0.0042 | 0.07691 | 0.06945 | -0.00944 |
| BJDX | Low | 0.0045 | 0.0807 | 0.073 | -0.0106 |
| SJY1 | Low | 0.0046 | 0.08295 | 0.07354 | -0.01504 |
| SJY4 | Low | 0.0045 | 0.08364 | 0.07448 | -0.01196 |
| SDSG | Low | 0.0046 | 0.07653 | 0.0692 | -0.00896 |
| SDWF | Low | 0.0049 | 0.0853 | 0.07234 | -0.0172 |
| SDST | Low | 0.0051 | 0.08602 | 0.07559 | -0.01366 |
| SCCD | Medium | 0.0051 | 0.09289 | 0.08516 | -0.00978 |
| SCDY | Medium | 0.0057 | 0.09775 | 0.08894 | -0.0105 |
| HNCS | Medium | 0.0051 | 0.08723 | 0.08153 | -0.00439 |
| GDZQ | Medium | 0.0056 | 0.09035 | 0.07977 | -0.01407 |
| GDSZ | Medium | 0.0055 | 0.09177 | 0.08623 | -0.00064 |
| YNBN | High | 0.0064 | 0.1061 | 0.09661 | -0.01195 |
| YNXS | High | 0.0063 | 0.10301 | 0.09755 | -0.00269 |
| HNSY | Low | 0.0047 | 0.07923 | 0.07181 | -0.00791 |
| HNSN | Low | 0.0050 | 0.07999 | 0.07127 | -0.01371 |

Note: Diversity level was classified based on the overall genetic diversity of all estimated parameters.

Abbreviations: *H*o, observed heterozygosity; *H*e, expected heterozygosity; *π*, nucleotide diversity; *F*_IS_, inbreeding coefficient.

**Table S4** Tests of homogeneity of variance (Levene test) as well as differences in CT_max_ and CCRT among populations (by Kruskal-Wallis (KW) tests) from different groupings.

| Methods | Populations | Levene statistic | Significance | KW statistic | P, asymptotic significance (2-sided test) |
| --- | --- | --- | --- | --- | --- |
| CT_max_ | All populations | 7.397 | <0.001 | 588.520 | <0.001 |
|  | Field group | 17.256 | <0.001 | 206.698 | <0.001 |
|  | Greenhouse group | 2.586 | 0.025 | 364.371 | <0.001 |
| CCRT | All populations | 16.328 | <0.001 | 130.162 | <0.001 |
|  | Field group | 28.330 | <0.001 | 102.427 | <0.001 |
|  | Greenhouse group | 9.224 | <0.001 | 33.494 | <0.001 |

**Table S5** P-values of Mann–Whitney U tests for pairwise differences in CT_max_ between populations for 11 populations of *Thrips palmi.* NS = non-significant. Note that all P values indicated as P<0.001 remain significant after Bonferroni correction.

**A: All populations**

|  | HNS2 | HNSN | GDZQ | YNBN | SCDY | SDWF | SDS2 | SDST | BJDX | BJFS |
| --- | --- | --- | --- | --- | --- | --- | --- | --- | --- | --- |
| HNSN | <0.001 |  |  |  |  |  |  |  |  |  |
| GDZQ | <0.001 | NS |  |  |  |  |  |  |  |  |
| YNBN | <0.001 | NS | NS |  |  |  |  |  |  |  |
| SCDY | <0.001 | <0.001 | <0.001 | <0.001 |  |  |  |  |  |  |
| SDWF | NS | NS | <0.001 | <0.001 | <0.001 |  |  |  |  |  |
| SDS2 | NS | NS | NS | <0.001 | <0.001 | NS |  |  |  |  |
| SDST | NS | NS | NS | <0.001 | <0.001 | NS | NS |  |  |  |
| BJDX | <0.001 | <0.001 | <0.001 | NS | NS | <0.001 | <0.001 | <0.001 |  |  |
| BJFS | <0.001 | <0.001 | <0.001 | NS | NS | <0.001 | <0.001 | <0.001 | NS |  |
| NMHS | <0.001 | <0.001 | <0.001 | <0.001 | NS | <0.001 | <0.001 | <0.001 | NS | <0.001 |

**B: Field group**

|  | HNS2 | HNSN | GDZQ | YNBN |
| --- | --- | --- | --- | --- |
| HNSN | <0.001 |  |  |  |
| GDZQ | <0.001 | NS |  |  |
| YNBN | <0.001 | NS | NS |  |
| SCDY | <0.001 | <0.001 | <0.001 | <0.001 |

**C: Greenhouse group**

|  | SDWF | SDS2 | SDST | BJDX | BJFS |
| --- | --- | --- | --- | --- | --- |
| SDS2 | NS |  |  |  |  |
| SDST | NS | NS |  |  |  |
| BJDX | <0.001 | <0.001 | <0.001 |  |  |
| BJFS | <0.001 | <0.001 | <0.001 | NS |  |
| NMHS | <0.001 | <0.001 | <0.001 | NS | <0.001 |

**Table S6** P-values of Mann–Whitney U test for the CCRT between populations in 11 populations of *Thrips palmi.* Note that all P values indicated as P<0.001 remain significant after Bonferroni correction.

**A: All populations**

|  | HNS2 | HNSN | GDZQ | YNBN | SCDY | SDWF | SDS2 | SDST | BJDX | BJFS |
| --- | --- | --- | --- | --- | --- | --- | --- | --- | --- | --- |
| HNSN | <0.001 |  |  |  |  |  |  |  |  |  |
| GDZQ | NS | NS |  |  |  |  |  |  |  |  |
| YNBN | <0.001 | NS | NS |  |  |  |  |  |  |  |
| SCDY | <0.001 | NS | <0.001 | <0.001 |  |  |  |  |  |  |
| SDWF | <0.001 | NS | NS | NS | NS |  |  |  |  |  |
| SDS2 | NS | NS | NS | NS | <0.001 | NS |  |  |  |  |
| SDST | <0.001 | NS | NS | NS | NS | NS | NS |  |  |  |
| BJDX | <0.001 | NS | NS | NS | NS | NS | NS | NS |  |  |
| BJFS | NS | <0.001 | NS | NS | <0.001 | NS | NS | <0.001 | NS |  |
| NMHS | <0.001 | NS | NS | NS | <0.001 | NS | NS | NS | NS | NS |

**B: Field group**

|  | HNS2 | HNST | GDZQ | YNBN |
| --- | --- | --- | --- | --- |
| HNST | <0.001 |  |  |  |
| GDZQ | <0.001 | NS |  |  |
| YNBN | <0.001 | NS | NS |  |
| SCDY | <0.001 | NS | <0.001 | <0.001 |

**C: Greenhouse group**

|  | SDWF | SDS2 | SDST | BJDX | BJFS |
| --- | --- | --- | --- | --- | --- |
| SDS2 | NS |  |  |  |  |
| SDST | NS | NS |  |  |  |
| BJDX | NS | NS | NS |  |  |
| BJFS | NS | NS | <0.001 | NS |  |
| NMHS | NS | NS | NS | NS | NS |

**Table S7** ANOVA and linear regression analysis of 19 bioclimatic variables and latitude on CT_max_ (mean for linear regression analysis) in 11 populations of *Thrips palmi.* The linear regression analyses were conducted with the geom_smooth R function.

|  |  | All populations | | | | | | Greenhouse group | | | | | | Field group | | |  |  |
| --- | --- | --- | --- | --- | --- | --- | --- | --- | --- | --- | --- | --- | --- | --- | --- | --- | --- | --- |
| **Explanatory** | **Df** | **Sum of squares** | **Mean square** | ***F*-value** | ***P*-value** | ***R*^2^ _adj_** | **Df** | **Sum of squares** | **Mean square** | ***F*-value** | ***P*-value** | ***R*^2^ _adj_** | **Df** | **Sum of squares** | **Mean square** | ***F*-value** | ***P*-value** | ***R*^2^ _adj_** |
| latitude | 1 | 3.19 | 3.19 | 2.3 | 0.164 | 0.11 | 1 | 7.36 | 7.36 | 231 | <0.001 | 0.98 | 1 | 4.76 | 4.76 | 4.8 | 0.116 | 0.49 |
| Residuals | 9 | 12.50 | 1.39 |  |  |  | 4 | 0.13 | 0.03 |  |  |  | 3 | 2.98 | 0.99 |  |  |  |
| altitude | 1 | 3.86 | 3.86 | 2.93 | 0.121 | 0.16 | 1 | 3.37 | 3.37 | 3.27 | 0.145 | 0.31 | 1 | 1.49 | 1.49 | 0.716 | 0.460 | <0.01 |
| Residuals | 9 | 11.80 | 1.31 |  |  |  | 4 | 4.12 | 1.03 |  |  |  | 3 | 6.25 | 2.08 |  |  |  |
| bio1 | 1 | 4.98 | 4.98 | 4.18 | 0.071 | 0.24 | 1 | 4.66 | 4.66 | 6.6 | 0.062 | 0.53 | 1 | 5.36 | 5.36 | 6.76 | 0.08 | 0.59 |
| Residuals | 9 | 10.70 | 1.19 |  |  |  | 4 | 2.83 | 0.71 |  |  |  | 3 | 2.38 | 0.79 |  |  |  |
| bio2 | 1 | 3.16 | 3.16 | 2.27 | 0.166 | 0.11 | 1 | 4.34 | 4.34 | 5.51 | 0.079 | 0.47 | 1 | 0.71 | 0.71 | 0.302 | 0.621 | <0.01 |
| Residuals | 9 | 12.50 | 1.39 |  |  |  | 4 | 3.15 | 0.79 |  |  |  | 3 | 7.03 | 2.34 |  |  |  |
| bio3 | 1 | 1.66 | 1.66 | 1.07 | 0.328 | <0.01 | 1 | 1.49 | 1.49 | 0.991 | 0.376 | <0.01 | 1 | 1.72 | 1.72 | 0.857 | 0.423 | <0.01 |
| Residuals | 9 | 14.00 | 1.56 |  |  |  | 4 | 6.00 | 1.50 |  |  |  | 3 | 6.02 | 2.01 |  |  |  |
| bio4 | 1 | 2.42 | 2.42 | 1.64 | 0.232 | 0.06 | 1 | 5.61 | 5.61 | 12 | 0.026 | 0.69 | 1 | 3.46 | 3.46 | 2.43 | 0.217 | 0.26 |
| Residuals | 9 | 13.30 | 1.47 |  |  |  | 4 | 1.87 | 0.47 |  |  |  | 3 | 4.28 | 1.43 |  |  |  |
| bio5 | 1 | 8.70 | 8.70 | 11.2 | 0.009 | 0.50 | 1 | 4.40 | 4.40 | 5.72 | 0.075 | 0.49 | 1 | 3.85 | 3.85 | 2.97 | 0.183 | 0.33 |
| Residuals | 9 | 6.99 | 0.78 |  |  |  | 4 | 3.08 | 0.77 |  |  |  | 3 | 3.89 | 1.30 |  |  |  |
| bio6 | 1 | 4.16 | 4.16 | 3.24 | 0.105 | 0.18 | 1 | 5.10 | 5.10 | 8.56 | 0.043 | 0.60 | 1 | 5.38 | 5.38 | 6.83 | 0.079 | 0.59 |
| Residuals | 9 | 11.50 | 1.28 |  |  |  | 4 | 2.38 | 0.60 |  |  |  | 3 | 2.36 | 0.79 |  |  |  |
| bio7 | 1 | 3.23 | 3.23 | 2.34 | 0.161 | 0.12 | 1 | 5.15 | 5.15 | 8.83 | 0.041 | 0.61 | 1 | 5.26 | 5.26 | 6.37 | 0.086 | 0.57 |
| Residuals | 9 | 12.50 | 1.38 |  |  |  | 4 | 2.33 | 0.58 |  |  |  | 3 | 2.48 | 0.83 |  |  |  |
| bio8 | 1 | 6.82 | 6.82 | 6.93 | 0.027 | 0.37 | 1 | 3.39 | 3.39 | 3.3 | 0.143 | 0.32 | 1 | 3.22 | 3.22 | 2.13 | 0.240 | 0.22 |
| Residuals | 9 | 8.87 | 0.99 |  |  |  | 4 | 4.10 | 1.02 |  |  |  | 3 | 4.52 | 1.21 |  |  |  |
| bio9 | 1 | 3.74 | 3.74 | 2.82 | 0.128 | 0.15 | 1 | 4.86 | 4.86 | 7.4 | 0.053 | 0.56 | 1 | 5.02 | 5.02 | 5.52 | 0.100 | 0.53 |
| Residuals | 9 | 12.00 | 1.33 |  |  |  | 4 | 2.63 | 0.66 |  |  |  | 3 | 2.72 | 0.91 |  |  |  |
| bio10 | 1 | 7.83 | 7.83 | 8.97 | 0.015 | 0.44 | 1 | 4.53 | 4.53 | 6.12 | 0.069 | 0.51 | 1 | 3.21 | 3.21 | 2.12 | 0.241 | 0.22 |
| Residuals | 9 | 7.86 | 0.87 |  |  |  | 4 | 2.96 | 0.74 |  |  |  | 3 | 4.53 | 1.51 |  |  |  |
| bio11 | 1 | 3.88 | 3.88 | 2.96 | 0.12 | 0.16 | 1 | 4.86 | 4.86 | 7.4 | 0.053 | 0.56 | 1 | 5.15 | 5.15 | 5.97 | 0.092 | 0.55 |
| Residuals | 9 | 11.80 | 1.31 |  |  |  | 4 | 2.63 | 0.66 |  |  |  | 3 | 2.89 | 0.86 |  |  |  |
| bio12 | 1 | 2.18 | 2.18 | 1.45 | 0.259 | 0.04 | 1 | 5.25 | 5.25 | 9.38 | 0.038 | 0.63 | 1 | 0.94 | 0.94 | 0.414 | 0.566 | <0.01 |
| Residuals | 9 | 13.50 | 1.50 |  |  |  | 4 | 2.24 | 0.56 |  |  |  | 3 | 6.80 | 2.27 |  |  |  |
| bio13 | 1 | 3.49 | 3.49 | 2.57 | 0.143 | 0.14 | 1 | 2.52 | 2.52 | 2.03 | 0.227 | 0.17 | 1 | 1.50 | 1.50 | 0.722 | 0.458 | <0.01 |
| Residuals | 9 | 12.20 | 1.36 |  |  |  | 4 | 4.97 | 1.24 |  |  |  | 3 | 6.24 | 2.08 |  |  |  |
| bio14 | 1 | 2.76 | 2.76 | 1.92 | 0.199 | 0.08 | 1 | 5.96 | 5.96 | 15.7 | 0.017 | 0.75 | 1 | 1.26 | 1.26 | 0.584 | 0.500 | <0.01 |
| Residuals | 9 | 12.90 | 1.44 |  |  |  | 4 | 1.52 | 0.38 |  |  |  | 3 | 6.48 | 2.16 |  |  |  |
| bio15 | 1 | 2.52 | 2.52 | 1.72 | 0.222 | 0.07 | 1 | 1.77 | 1.77 | 1.24 | 0.328 | 0.05 | 1 | 2.09 | 2.09 | 1.11 | 0.370 | 0.03 |
| Residuals | 9 | 13.20 | 1.46 |  |  |  | 4 | 5.72 | 1.43 |  |  |  | 3 | 5.65 | 1.88 |  |  |  |
| bio16 | 1 | 2.10 | 2.10 | 1.39 | 0.268 | 0.04 | 1 | 1.96 | 1.96 | 1.42 | 0.299 | 0.08 | 1 | 1.09 | 1.09 | 0.491 | 0.534 | <0.01 |
| Residuals | 9 | 13.60 | 1.51 |  |  |  | 4 | 5.52 | 1.38 |  |  |  | 3 | 6.65 | 2.22 |  |  |  |
| bio17 | 1 | 2.93 | 2.93 | 2.06 | 0.185 | 0.10 | 1 | 6.50 | 6.50 | 26.4 | 0.007 | 0.84 | 1 | 1.42 | 1.42 | 0.673 | 0.472 | <0.01 |
| Residuals | 9 | 12.80 | 1.42 |  |  |  | 4 | 0.98 | 0.25 |  |  |  | 3 | 6.32 | 2.11 |  |  |  |
| bio18 | 1 | 0.008 | 0.01 | 0.00459 | 0.947 | <0.01 | 1 | 1.95 | 1.95 | 1.41 | 0.301 | 0.08 | 1 | 1.84 | 1.84 | 0.933 | 0.405 | <0.01 |
| Residuals | 9 | 15.70 | 1.74 |  |  |  | 4 | 5.53 | 1.38 |  |  |  | 3 | 5.90 | 1.97 |  |  |  |
| bio19 | 1 | 2.85 | 2.85 | 2 | 0.191 | 0.09 | 1 | 6.50 | 6.50 | 26.4 | 0.007 | 0.84 | 1 | 1.66 | 1.66 | 0.818 | 0.432 | <0.01 |
| Residuals | 9 | 12.80 | 1.43 |  |  |  | 4 | 0.98 | 0.25 |  |  |  | 3 | 6.08 | 2.03 |  |  |  |

**Table S8** ANOVA and linear regression analysis of 19 bioclimatic variables and latitude on CCRT (mean for linear regression analysis) in 11 populations of *Thrips palmi.* The linear regression analyses were conducted with the geom_smooth R function.

|  |  | All populations | | | | | | Greenhouse group | | | | | | Field group | | |  |  |
| --- | --- | --- | --- | --- | --- | --- | --- | --- | --- | --- | --- | --- | --- | --- | --- | --- | --- | --- |
| **Explanatory** | **Df** | **Sum of squares** | **Mean square** | ***F*-value** | ***P*-value** | ***R*^2^ _adj_** | **Df** | **Sum of squares** | **Mean square** | ***F*-value** | ***P*-value** | ***R*^2^ _adj_** | **Df** | **Sum of squares** | **Mean square** | ***F*-value** | ***P*-value** | ***R*^2^ _adj_** |
| latitude | 1 | 0.94 | 0.94 | 0.298 | 0.598 | <0.01 | 1 | 1.58 | 1.58 | 1.050 | 0.363 | 0.01 | 1 | 15.40 | 15.40 | 7.390 | 0.073 | 0.61 |
| Residuals | 9 | 28.40 | 3.15 |  |  |  | 4 | 5.98 | 1.50 |  |  |  | 3 | 6.26 | 2.09 |  |  |  |
| altitude | 1 | 1.44 | 1.44 | 0.466 | 0.512 | <0.01 | 1 | 0.03 | 0.03 | 0.016 | 0.905 | <0.01 | 1 | 3.08 | 3.08 | 0.497 | 0.532 | <0.01 |
| Residuals | 9 | 27.90 | 3.10 |  |  |  | 4 | 7.53 | 1.88 |  |  |  | 3 | 18.60 | 6.19 |  |  |  |
| bio1 | 1 | 1.44 | 1.44 | 0.466 | 0.512 | <0.01 | 1 | 0.13 | 0.13 | 0.072 | 0.802 | <0.01 | 1 | 15.20 | 15.20 | 7.000 | 0.077 | 0.60 |
| Residuals | 9 | 27.90 | 3.10 |  |  |  | 4 | 7.43 | 1.86 |  |  |  | 3 | 6.49 | 2.16 |  |  |  |
| bio2 | 1 | 0.05 | 0.05 | 0.017 | 0.900 | <0.01 | 1 | 0.01 | 0.01 | 0.007 | 0.939 | <0.01 | 1 | 0.59 | 0.59 | 0.084 | 0.791 | <0.01 |
| Residuals | 9 | 29.30 | 3.25 |  |  |  | 4 | 7.55 | 1.89 |  |  |  | 3 | 21.10 | 7.02 |  |  |  |
| bio3 | 1 | 1.77 | 1.77 | 0.579 | 0.466 | <0.01 | 1 | 0.62 | 0.62 | 0.360 | 0.581 | <0.01 | 1 | 5.00 | 5.00 | 0.900 | 0.413 | <0.01 |
| Residuals | 9 | 27.50 | 3.06 |  |  |  | 4 | 6.94 | 1.73 |  |  |  | 3 | 16.70 | 5.55 |  |  |  |
| bio4 | 1 | 0.44 | 0.44 | 0.136 | 0.721 | <0.01 | 1 | 0.31 | 0.31 | 0.172 | 0.699 | <0.01 | 1 | 9.66 | 9.66 | 2.410 | 0.218 | 0.26 |
| Residuals | 9 | 28.90 | 3.21 |  |  |  | 4 | 7.25 | 1.81 |  |  |  | 3 | 12.00 | 4.00 |  |  |  |
| bio5 | 1 | 4.11 | 4.11 | 1.470 | 0.256 | 0.04 | 1 | 0.26 | 0.26 | 0.144 | 0.723 | <0.01 | 1 | 13.20 | 13.20 | 4.720 | 0.118 | 0.48 |
| Residuals | 9 | 25.20 | 2.80 |  |  |  | 4 | 7.30 | 1.82 |  |  |  | 3 | 8.42 | 2.81 |  |  |  |
| bio6 | 1 | 0.80 | 0.80 | 0.253 | 0.627 | <0.01 | 1 | 0.11 | 0.11 | 0.059 | 0.820 | <0.01 | 1 | 13.50 | 13.50 | 4.960 | 0.112 | 0.50 |
| Residuals | 9 | 28.50 | 3.17 |  |  |  | 4 | 7.45 | 1.86 |  |  |  | 3 | 8.17 | 2.72 |  |  |  |
| bio7 | 1 | 0.49 | 0.49 | 0.152 | 0.706 | <0.01 | 1 | 0.06 | 0.06 | 0.033 | 0.866 | <0.01 | 1 | 12.10 | 12.10 | 3.810 | 0.146 | 0.41 |
| Residuals | 9 | 28.80 | 3.20 |  |  |  | 4 | 7.50 | 1.87 |  |  |  | 3 | 9.54 | 3.18 |  |  |  |
| bio8 | 1 | 1.61 | 1.61 | 0.524 | 0.488 | <0.01 | 1 | 0.10 | 0.10 | 0.056 | 0.825 | <0.01 | 1 | 9.49 | 9.49 | 2.340 | 0.223 | 0.25 |
| Residuals | 9 | 27.70 | 3.08 |  |  |  | 4 | 7.46 | 1.86 |  |  |  | 3 | 12.20 | 4.05 |  |  |  |
| bio9 | 1 | 1.02 | 1.02 | 0.326 | 0.582 | <0.01 | 1 | 0.16 | 0.16 | 0.087 | 0.783 | <0.01 | 1 | 15.30 | 15.30 | 7.220 | 0.075 | 0.61 |
| Residuals | 9 | 28.30 | 3.14 |  |  |  | 4 | 7.40 | 1.85 |  |  |  | 3 | 6.36 | 2.12 |  |  |  |
| bio10 | 1 | 2.67 | 2.67 | 0.904 | 0.367 | <0.01 | 1 | 0.12 | 0.12 | 0.065 | 0.811 | <0.01 | 1 | 8.40 | 8.40 | 1.900 | 0.262 | 0.18 |
| Residuals | 9 | 26.60 | 2.96 |  |  |  | 4 | 7.44 | 1.86 |  |  |  | 3 | 13.30 | 4.42 |  |  |  |
| bio11 | 1 | 0.94 | 0.94 | 0.297 | 0.599 | <0.01 | 1 | 0.16 | 0.16 | 0.087 | 0.783 | <0.01 | 1 | 14.30 | 14.30 | 5.890 | 0.094 | 0.55 |
| Residuals | 9 | 28.40 | 3.15 |  |  |  | 4 | 7.40 | 1.85 |  |  |  | 3 | 7.31 | 2.44 |  |  |  |
| bio12 | 1 | 0.70 | 0.70 | 0.219 | 0.651 | <0.01 | 1 | 0.75 | 0.75 | 0.438 | 0.544 | <0.01 | 1 | 10.40 | 10.40 | 2.790 | 0.193 | 0.31 |
| Residuals | 9 | 28.60 | 3.18 |  |  |  | 4 | 6.81 | 1.70 |  |  |  | 3 | 11.20 | 3.74 |  |  |  |
| bio13 | 1 | 0.43 | 0.42 | 0.133 | 0.723 | <0.01 | 1 | 0.12 | 0.12 | 0.065 | 0.811 | <0.01 | 1 | 11.70 | 11.70 | 3.540 | 0.156 | 0.39 |
| Residuals | 9 | 28.90 | 3.21 |  |  |  | 4 | 7.44 | 1.86 |  |  |  | 3 | 9.93 | 3.31 |  |  |  |
| bio14 | 1 | 2.36 | 2.36 | 0.787 | 0.398 | <0.01 | 1 | 3.06 | 3.06 | 2.720 | 0.174 | 0.26 | 1 | 10.30 | 10.30 | 2.750 | 0.196 | 0.30 |
| Residuals | 9 | 27.00 | 2.99 |  |  |  | 4 | 4.50 | 1.12 |  |  |  | 3 | 11.30 | 3.77 |  |  |  |
| bio15 | 1 | 0.02 | 0.02 | 0.007 | 0.935 | <0.01 | 1 | 1.41 | 1.41 | 0.917 | 0.392 | <0.01 | 1 | 13.50 | 13.50 | 4.990 | 0.112 | 0.50 |
| Residuals | 9 | 29.30 | 3.25 |  |  |  | 4 | 6.15 | 1.54 |  |  |  | 3 | 8.13 | 2.71 |  |  |  |
| bio16 | 1 | 0.46 | 0.46 | 0.143 | 0.714 | <0.01 | 1 | 0.04 | 0.04 | 0.022 | 0.890 | <0.01 | 1 | 10.50 | 10.50 | 2.800 | 0.193 | 0.31 |
| Residuals | 9 | 28.80 | 3.21 |  |  |  | 4 | 7.52 | 1.88 |  |  |  | 3 | 11.20 | 3.73 |  |  |  |
| bio17 | 1 | 2.22 | 2.22 | 0.739 | 0.412 | <0.01 | 1 | 2.67 | 2.67 | 2.180 | 0.214 | 0.19 | 1 | 10.40 | 10.40 | 2.790 | 0.193 | 0.31 |
| Residuals | 9 | 27.10 | 3.01 |  |  |  | 4 | 4.89 | 1.22 |  |  |  | 3 | 11.20 | 3.74 |  |  |  |
| bio18 | 1 | 0.67 | 0.67 | 0.209 | 0.658 | <0.01 | 1 | 0.04 | 0.04 | 0.022 | 0.890 | <0.01 | 1 | 0.59 | 0.59 | 0.083 | 0.792 | <0.01 |
| Residuals | 9 | 28.60 | 3.18 |  |  |  | 4 | 7.52 | 1.88 |  |  |  | 3 | 21.10 | 7.02 |  |  |  |
| bio19 | 1 | 2.62 | 2.62 | 0.885 | 0.371 | <0.01 | 1 | 2.67 | 2.67 | 2.180 | 0.214 | 0.19 | 1 | 11.50 | 11.50 | 3.420 | 0.162 | 0.38 |
| Residuals | 9 | 26.70 | 2.96 |  |  |  | 4 | 4.89 | 1.22 |  |  |  | 3 | 10.10 | 3.37 |  |  |  |

**Table S9** Identified gene functions potentially related to heat stress response in each analysis.

| Method, population | Actin | Microtube | HSP | Oxidoreductase |
| --- | --- | --- | --- | --- |
| kNN, Field group | Y | Y | Y | Y |
| kNN, Greenhouse group | - | Y | Y | Y |
| *F*_ST_, HNSN vs SCDY | Y | - | Y | Y |
| *F*_ST_, YNBN vs SCDY | Y | Y | Y | Y |
| *F*_ST_, BJDX vs SDS1 | Y | Y | Y | Y |
| *F*_ST_, NMHS vs SDS1 | *-* | Y | Y | Y |
| *F*_ST_, BJDX vs SDS3 | Y | Y | Y | Y |
| *F*_ST_, NMHS vs SDS3 | *-* | Y | Y | Y |
| *F*_ST_, SJY1 vs SJY4 | Y | Y | - | Y |
| *F*_ST_, High vs low CT_max_ individuals | Y | Y | Y | Y |
| LFMM, 4 field populations, CT_max_ | Y | Y | Y | Y |
| LFMM, 4 field populations, bio05 | Y | Y | Y | Y |
| LFMM, 8 field populations, bio05 | Y | Y |  | Y |
| LFMM, 4 greenhouse populations, CT_max_ | Y | Y | Y | Y |
| LFMM, 4 greenhouse populations, CT_max_, bio05 | Y | Y | Y | Y |
| LFMM, 6 greenhouse populations, CT_max_, bio05 | Y | Y | Y | Y |

Y, presence of the gene function; -, no corresponding gene function.

**Table S10** Gene number identified by kNN or *F*_ST_ in each pairwise comparison. The numbers of genes identified in each analysis are indicated in the leading diagonal (number of identified genes /(number of identified genes potentially heat stress response, cytoskeletal organization, and oxidation-reduction). Each cell in the lower triangle shows the number of overlapping genes /(number of overlapping genes associated with heat stress response, cytoskeletal organization, and oxidation-reduction). _F, Field group; _G, Greenhouse group; F_ST__LH, F_ST_ between low and high CT_max_ individuals in a population.

| Method | kNN_F | kNN_G | *F*_ST_, HNSN vs SCDY | *F*_ST_, YNBN vs SCDY | *F*_ST_, BJDX vs SDS1 | *F*_ST_, NMHS vs SDS1 | *F*_ST_, BJDX vs SDS3 | *F*_ST_, NMHS vs SDS3 | *F*_ST_, SJY1 vs SJY4 | *F*_ST__LH |
| --- | --- | --- | --- | --- | --- | --- | --- | --- | --- | --- |
| kNN_F | 720/29 |  |  |  |  |  |  |  |  |  |
| kNN_G | 85/0 | 652/33 |  |  |  |  |  |  |  |  |
| *F*_ST_, HNSN vs SCDY | 130/0 | 62/1 | 707/12 |  |  |  |  |  |  |  |
| F_ST_, YNBN vs SCDY | 120/0 | 32/1 | 192/4 | 949/13 |  |  |  |  |  |  |
| F_ST_, BJDX vs SDS1 | 65/0 | 36/4 | 178/2 | 178/2 | 1323/28 |  |  |  |  |  |
| F_ST_, NMHS vs SDS1 | 22/0 | 17/0 | 33/1 | 39/0 | 68/0 | 596/14 |  |  |  |  |
| F_ST_, BJDX vs SDS3 | 18/0 | 46/2 | 57/0 | 101/1 | 71/1 | 15/0 | 584/21 |  |  |  |
| F_ST_, NMHS vs SDS3 | 9/0 | 17/0 | 34/0 | 37/0 | 63/0 | 52/0 | 124/2 | 310/11 |  |  |
| F_ST_, SJY1 vs SJY4 | 26/0 | 22/1 | 52/1 | 111/1 | 131/3 | 15/0 | 33/1 | 21/0 | 574/34 |  |
| F_ST__LH | 12/9 | 0 | 11/0 | 64/0 | 62/0 | 34/4 | 13/0 | 26/0 | 41/0 | 466/19 |

**Table S11** Overlap of gene number identified by LFMM in each pairwise comparison. The numbers of genes identified in each analysis are indicated before the dividing line and gene numbers potentially related to the functions heat stress response, cytoskeletal organization, and oxidation-reduction are listed after the dividing line. As gene functions instead of genes identified were consistent between population groups and analyses, we only list shared genes potentially related to heat stress responses as above. FP, populations from Field group; GP, populations from greenhouse group.

| LFMM analysis | 4_FP, CT_max_ | 4_FP, bio05 | 8_FP, bio05 | 4_GP, bio05 | 4_GP, CT_max_ | 6_GP, bio05 |
| --- | --- | --- | --- | --- | --- | --- |
| 4_FP, CT_max_ | 1231/52 |  |  |  |  |  |
| 4_FP, bio05 | 620/48 | 922/93 |  |  |  |  |
| 8_FP, bio05 | 160/4 | 176/8 | 682/42 |  |  |  |
| 4_GP, bio05 | 19/0 | 21/0 | 10/2 | 793/36 |  |  |
| 4_GP, CT_max_ | 20/1 | 18/1 | 12/2 | 763/34 | 799/43 |  |
| 6_GP, bio05 | 35/1 | 39/2 | 16/4 | 752/34 | 735/39 | 811/44 |

**Table S12** Number of outlier genes located on the inverted chromosome regions

| Chromosome | chr03 | chr05 | chr14 |
| --- | --- | --- | --- |
| Length of inverted region (kb) | 667 | 1130 | 764 |
| Number of genes on inverted region | 16 | 87 | 66 |
| KNN_Field_group | 1 | 37 | 0 |
| KNN_Greenhouse_group | 0 | 39 | 0 |
| High_low_CTmax | 0 | 13 | 1 |
| SJY1 vs SJY4 | 0 | 1 | 1 |
| HNSN vs SCDY | 5 | 71 | 1 |
| YNBN vs SCDY | 1 | 0 | 5 |
| BJDX vs SDS1 | 0 | 4 | 0 |
| BJDX vs SDS3 | 0 | 1 | 0 |
| NMHS vs SDS1 | 0 | 0 | 0 |
| NMHS vs SDS3 | 0 | 0 | 0 |
| LFMM, 4 field populations, bio05 | 5 | 76 | 3 |
| LFMM, 4 greenhouse populations, bio05 | 0 | 0 | 0 |
| LFMM, 4 field populations, CTmax | 3 | 76 | 0 |
| LFMM, 4 greenhouse populations, CTmax | 0 | 0 | 0 |
| LFMM, 8 field populations, bio05 | 3 | 57 | 1 |
| LFMM, 6 field populations, bio05 | 0 | 3 | 0 |
| Number of genes identified as outliers in all analyses | 8 | 77 | 11 |

**Table S13** Outliers genes on inverted regions of chr3, chr5 and chr14. Five genes related to oxidation-reduction on the inverted regions of chr5 were shown in red.

| chr3 |  |  |
| --- | --- | --- |
| rna-XM_034374679.1 | Catalyzes the hydroxylation of the N(6)-(4-aminobutyl)- L-lysine intermediate to form hypusine | DOHH |
| rna-XM_034375030.1 | Protein of unknown function (DUF3421) | - |
| rna-XM_034375405.1 | Phospholipase A2 | - |
| rna-XM_034375406.1 | Phospholipase A2 | - |
| rna-XM_034375408.1 | It is involved in signal transduction | - |
| rna-XM_034375592.1 | Produces nitric oxide (NO) | NOS1 |
| rna-XM_034375593.1 | Domain of unknown function (DUF4464) | C4orf22 |
| rna-XM_034375597.1 | Domain first found in C1r, C1s, uEGF, and bone morphogenetic protein. | - |
| Chr5 |  |  |
| rna-XM_034378369.1 | Receptor binding. It is involved in the biological process described with regulation of cell migration | LAMA2 |
| rna-XM_034377811.1 | Piezo-type mechanosensitive ion channel component | - |
| rna-XM_034377812.1 | Piezo-type mechanosensitive ion channel component | - |
| rna-XM_034377813.1 | Piezo-type mechanosensitive ion channel component | - |
| rna-XM_034377814.1 | Piezo-type mechanosensitive ion channel component | - |
| rna-XM_034377815.1 | Piezo-type mechanosensitive ion channel component | - |
| rna-XM_034377816.1 | Piezo-type mechanosensitive ion channel component | - |
| rna-XM_034377818.1 | Piezo-type mechanosensitive ion channel component | - |
| rna-XM_034377853.1 | Tetratricopeptide repeat protein 21B-like | TTC21B |
| rna-XM_034377854.1 | GTP binding | GTPBP2 |
| rna-XM_034377855.1 | GTP binding | GTPBP2 |
| rna-XM_034377856.1 | Trypsin | - |
| rna-XM_034377857.1 | DNA/RNA non-specific endonuclease | ENDOG |
| rna-XM_034377859.1 | GCN5-related N-acetyl-transferase | NATD1 |
| rna-XM_034377861.1 | GCN5-related N-acetyl-transferase | NATD1 |
| rna-XM_034377975.1 | Belongs to the peptidase S1 family | - |
| rna-XM_034377976.1 | Belongs to the peptidase S1 family | - |
| rna-XM_034377977.1 | Oxidoreductase activity. It is involved in the biological process described with oxidation-reduction process | PAOX |
| rna-XM_034377978.1 | assists the folding of proteins upon ATP hydrolysis | cct7 |
| rna-XM_034377983.1 | protein serine threonine kinase activity. It is involved in protein phosphorylation | SIK3 |
| rna-XM_034377984.1 | protein serine threonine kinase activity. It is involved in protein phosphorylation | SIK3 |
| rna-XM_034377985.1 | protein serine threonine kinase activity. It is involved in protein phosphorylation | SIK3 |
| rna-XM_034378045.1 | Immunoglobulin like | PRTG |
| rna-XM_034378046.1 | NADH dehydrogenase ubiquinone | NDUFB8 |
| rna-XM_034378047.1 | PET assembly of cytochrome c oxidase, mitochondrial | PET117 |
| rna-XM_034378090.1 | Juvenile hormone binding protein domains in insects. | to |
| rna-XM_034378147.1 | Rieske [2Fe-2S] domain | nvd |
| rna-XM_034378148.1 | Rieske [2Fe-2S] domain | nvd |
| rna-XM_034378193.1 | Spondin_N | SPON2 |
| rna-XM_034378218.1 | Sequence-specific DNA binding. It is involved in regulation of transcription, DNA-templated | NKX6-1 |
| rna-XM_034378289.1 | Domain of unknown function (DUF1989) | - |
| rna-XM_034378290.1 | Domain of unknown function (DUF1989) | - |
| rna-XM_034378368.1 | Ionotropic glutamate receptor-invertebrate | - |
| rna-XM_034378370.1 | Homeodomain | PRRX2 |
| rna-XM_034378371.1 | Ammonium transporter | amt-3 |
| rna-XM_034378382.1 | transferase activity, transferring acyl groups other than amino-acyl groups | - |
| rna-XM_034378406.1 | EF-hand domain pair | NUCB2 |
| rna-XM_034378495.1 | Zinc ion binding | TANC2 |
| rna-XM_034378589.1 | Ligand binding domain of hormone receptors | Hr4 |
| rna-XM_034378710.1 | Phosphotransferase activity, alcohol group as acceptor | PI4K2B |
| rna-XM_034378712.1 | BAR | SH3GLB1 |
| rna-XM_034378713.1 | BAR | SH3GLB1 |
| rna-XM_034378714.1 | Vacuolar ATPase is responsible for acidifying a variety of intracellular compartments in eukaryotic cells | ATP6V0E1 |
| rna-XM_034378723.1 | Low-density lipoprotein receptor domain class A | - |
| rna-XM_034378762.1 | Phosphoglycerate mutase family | PGAM2 |
| rna-XM_034378763.1 | Phosphoglycerate mutase family | PGAM2 |
| rna-XM_034378764.1 | ADP-ribosylation factor family | - |
| rna-XM_034378765.1 | Ndr family | - |
| rna-XM_034378766.1 | Ndr family | - |
| rna-XM_034378767.1 | Ndr family | - |
| rna-XM_034378780.1 | UTP glucose-1-phosphate uridylyltransferase activity. It is involved in UDP-glucose metabolic process | UGP2 |
| rna-XM_034378781.1 | UTP glucose-1-phosphate uridylyltransferase activity. It is involved in UDP-glucose metabolic process | UGP2 |
| rna-XM_034378841.1 | sequence-specific DNA binding. It is involved in regulation of transcription, DNA-templated | - |
| rna-XM_034378914.1 | It is involved in the biological process described with nucleotide-excision repair | ERCC2 |
| rna-XM_034378979.1 | heme binding. It is involved in the biological process described with oxidation-reduction process | CYP307A1 |
| rna-XM_034379187.1 | sequence-specific DNA binding. It is involved regulation of transcription, DNA-templated | ATF6 |
| rna-XM_034379188.1 | sequence-specific DNA binding. It is involved regulation of transcription, DNA-templated | ATF6 |
| rna-XM_034379189.1 | sequence-specific DNA binding. It is involved regulation of transcription, DNA-templated | ATF6 |
| rna-XM_034379190.1 | protein dimerization activity | BTRC |
| rna-XM_034379191.1 | protein dimerization activity | BTRC |
| rna-XM_034379194.1 | exodeoxyribonuclease III activity | RAD1 |
| rna-XM_034379195.1 | exodeoxyribonuclease III activity | RAD1 |
| rna-XM_034379196.1 | PA domain | PRADC1 |
| rna-XM_034379197.1 | Sulfiredoxin activity. It is involved oxidation-reduction process | SRXN1 |
| rna-XM_034379198.1 | Sulfiredoxin activity. It is involved oxidation-reduction process | SRXN1 |
| rna-XM_034379199.1 | Sulfiredoxin activity. It is involved oxidation-reduction process | SRXN1 |
| rna-XM_034379315.1 | Guanine nucleotide exchange factor for Ras-like GTPases; N-terminal motif | SOS1 |
| rna-XM_034379316.1 | It is involved ribosome biogenesis | NOC4L |
| rna-XM_034379317.1 | GTP binding. It is involved protein transport | RAB11A |
| rna-XM_034379318.1 | GTP binding. It is involved protein transport | RAB11A |
| rna-XM_034379319.1 | GTP binding. It is involved in the biological process described with protein transport | RAB11A |
| rna-XM_034379549.1 | Belongs to the ligand-gated ion channel (TC 1.A.9) family | GLRA3 |
| rna-XM_034379550.1 | Belongs to the ligand-gated ion channel (TC 1.A.9) family | GLRA3 |
| rna-XM_034379551.1 | Belongs to the ligand-gated ion channel (TC 1.A.9) family | GLRA3 |
| rna-XM_034379552.1 | Belongs to the ligand-gated ion channel (TC 1.A.9) family | GLRA3 |
| rna-XM_034379553.1 | LSM domain | LSM1 |
| rna-XM_034379554.1 | LSM domain | LSM1 |
| Chr14 |  |  |
| rna-XM_034396777.1 | Matrixin | MMP15 |
| rna-XM_034396775.1 | Matrixin | MMP15 |
| rna-XM_034396776.1 | Matrixin | MMP15 |
| rna-XM_034396779.1 | Pyridine nucleotide-disulphide oxidoreductase, dimerisation domain | TXNRD2 |
| rna-XM_034396778.1 | Pyridine nucleotide-disulphide oxidoreductase, dimerisation domain | TXNRD2 |
| rna-XM_034396613.1 | C2H2-type zinc finger | SALL1 |
| rna-XM_034395819.1 | BTG family | TOB1 |
| rna-XM_034396247.1 | 7 transmembrane receptor (Secretin family) | - |
| rna-XM_034396168.1 | Belongs to the sodium neurotransmitter symporter (SNF) (TC 2.A.22) family | - |
| rna-XM_034396170.1 | Belongs to the sodium neurotransmitter symporter (SNF) (TC 2.A.22) family | - |
| rna-XM_034396169.1 | Belongs to the sodium neurotransmitter symporter (SNF) (TC 2.A.22) family | - |


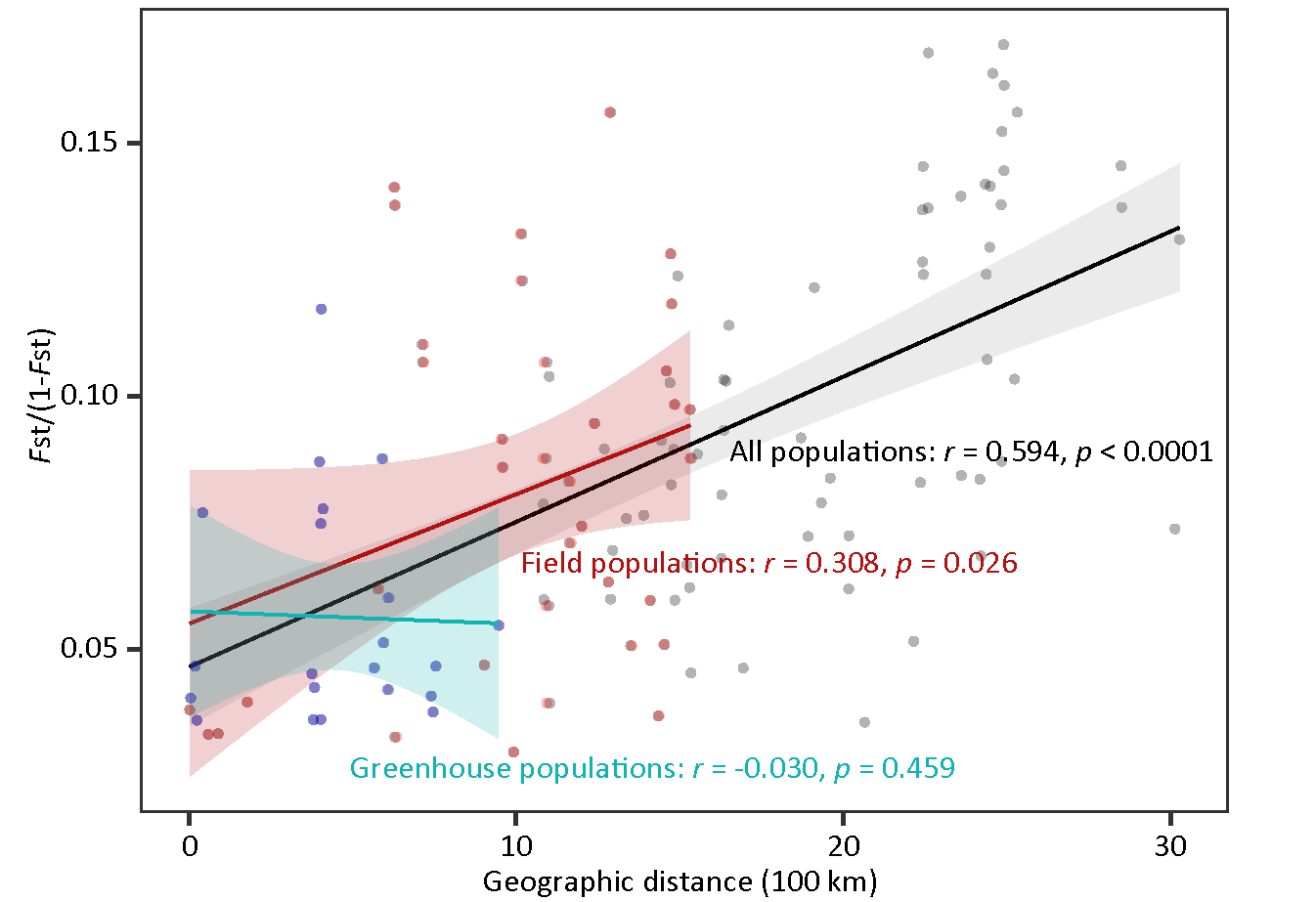


**Fig. S1** Plots of isolation-by-distance for all populations, the Field group, and the Greenhouse group of *Thrips palmi*. Regression lines and confidence intervals around the regression lines are shown. The *r* and *p* values from Mantel tests are indicated.


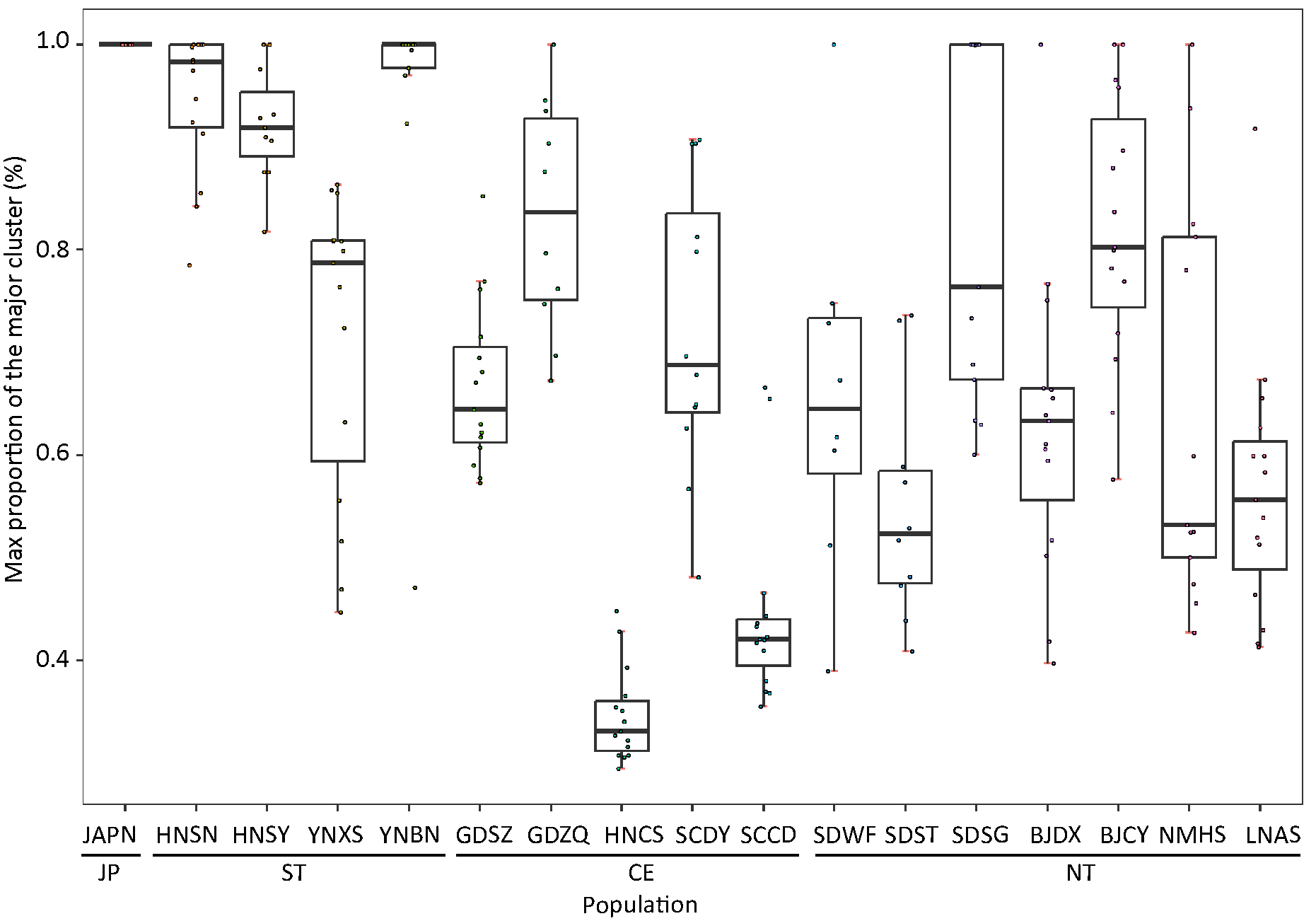
**Fig. S2** Level of genetic admixture for individuals from each population. The proportion of the major genetic clusters to which populations belonged when k = 8 in the ADMIXTURE analysis is provided. Box plots show the upper quartile, lower quartile and medians of maximum proportion of the major cluster.


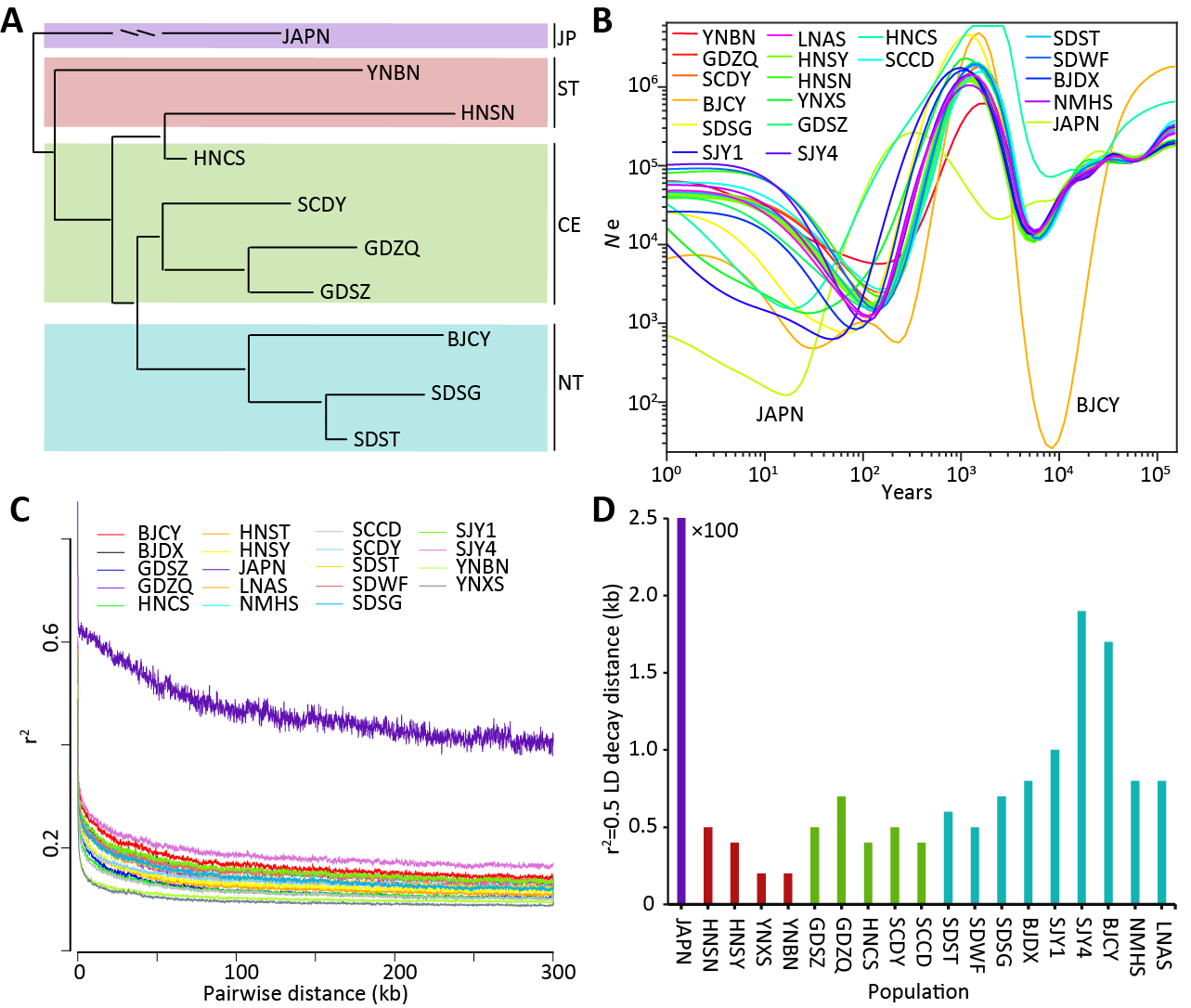


**Fig. S3** (A) Demographic history of four *T. palmi* groups estimated by KIMTREE, with the four colors denoting four groups identified by ADMIXTURE analysis (k = 4). (B) Estimates of the effective population size, *N*e, for each of the *T. palmi* populations using SMC++ inferences with 50 replicated runs. We set the generation time (g) as 0.1 years and the mutation rate (µ) as 8.4 × 10^−9^ per site per generation. *N*_e_ frequently fluctuated during the past 10,000 years, with two bottleneck events occurring around the last glacial maximum (LGM, 10,000–20,000 years ago) and 100 years ago and increasing in the past 100 to ten years. The largest difference in *N*_e_ was between populations from China and Japan. In China, BJCY and HNCS had distinct *N*_e_ patterns from those of other populations. (C) Decay of linkage disequilibrium of *T. palmi* populations measured by r^2^. Different colored lines show different populations. All populations showed similar patterns of effective population size (*N*_e_). (D) Distribution of LD decay distance at r^2^=0.5 for each population and the color is associated with corresponding groups. The population from Japan had very high linkage disequilibrium (LD) with a half LD decay (*r^2^* = 0.5) distance reaching 25.12 kb. In populations from China, the half LD decay distance ranged from 0.2 to 1.9 kb, with an average value of 1.26 kb. Northern greenhouse populations had a higher level of linkage than southern populations.


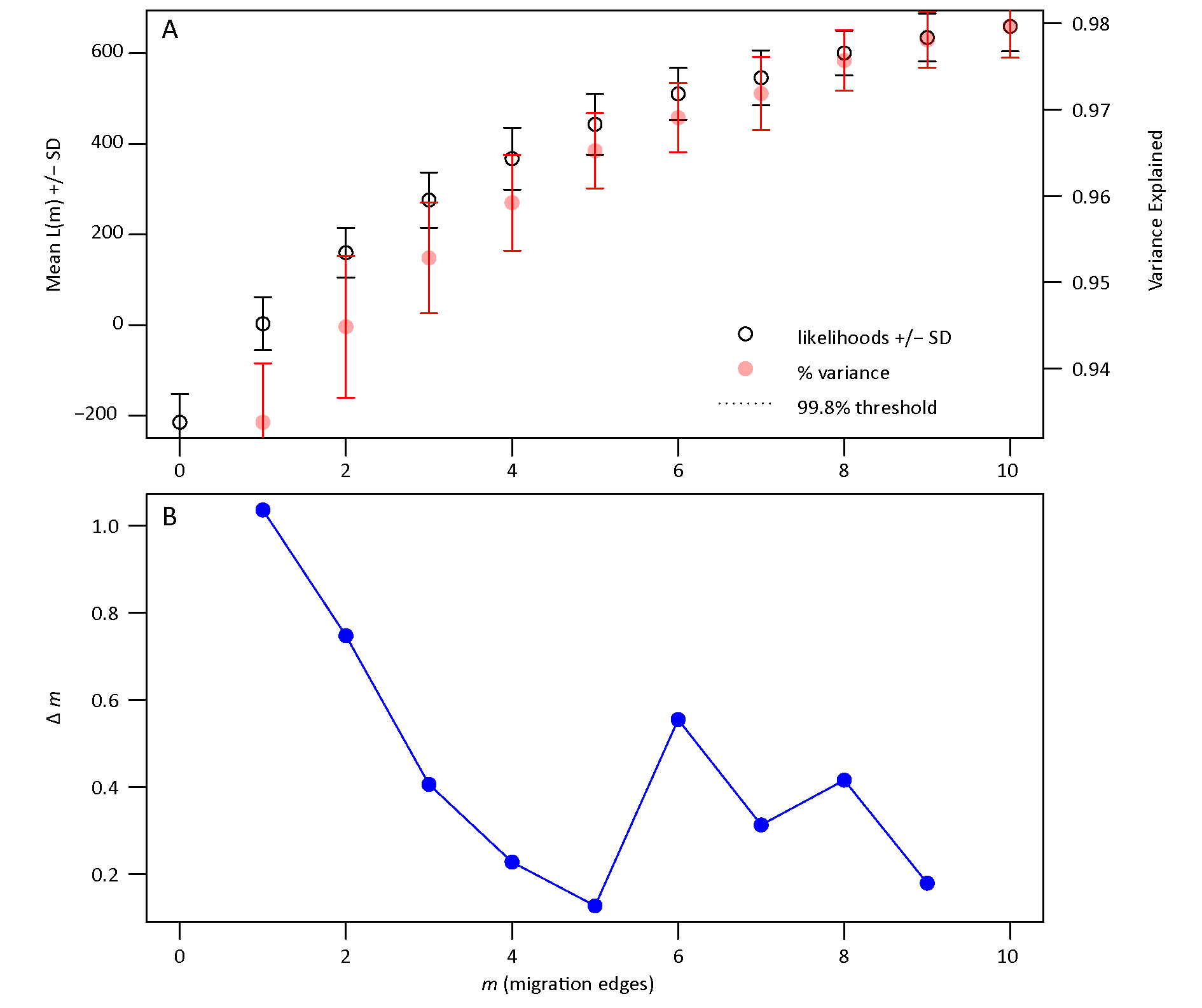


**Fig. S4** The best estimated number of migration events (*m*) detected by the Evanno method embedded in R package *optM*. (A) Mean log-likelihood and proportion of the variance explained by the tree model over 100 replicates for 0-10 migration nodes. (B) Migration nodes. The best number of migration events is 1.


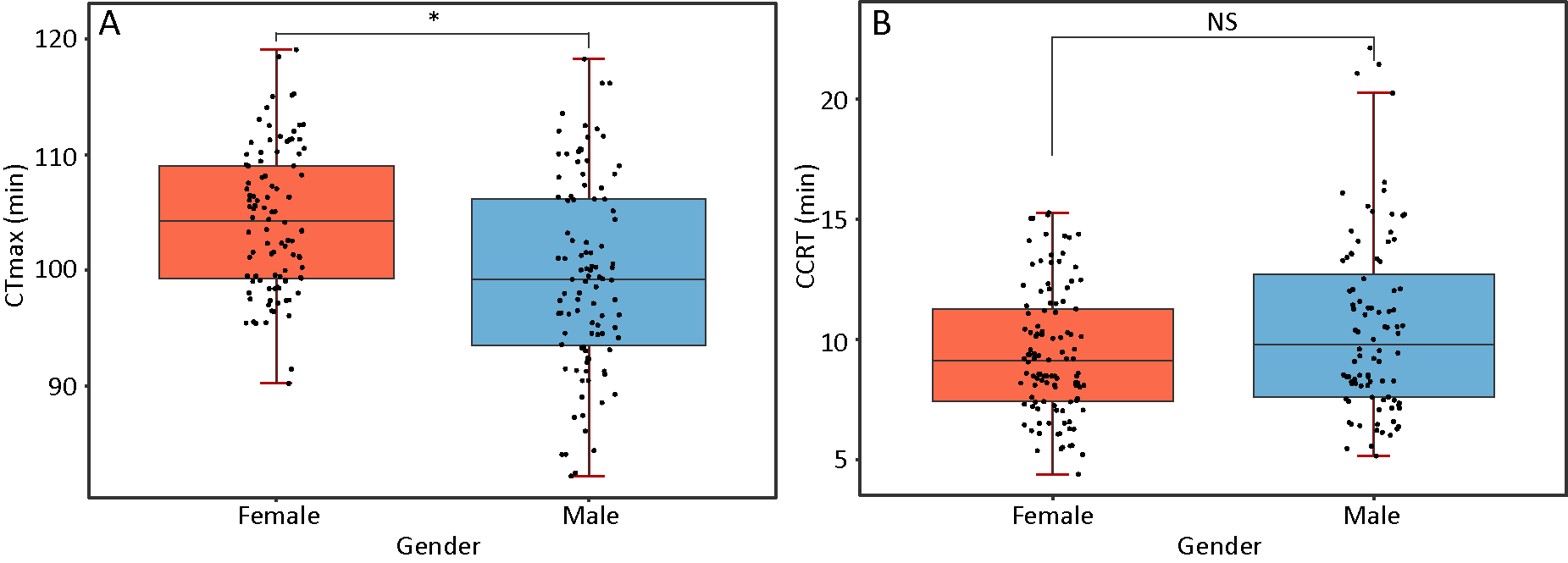


**Fig. S5** Boxplots showing the difference in CT_max_ (A) and CCRT (B) between male and female adults of *Thrips* *palmi* from the population comparisons. The heat tolerance of females was significantly higher than that of males, but there was no significant difference in cold tolerance between females and males. A Mann-Whitney test was used to analyze the difference in CT_max_ and CCRT between male and female adults. NS = not significant; * P < 0.05.


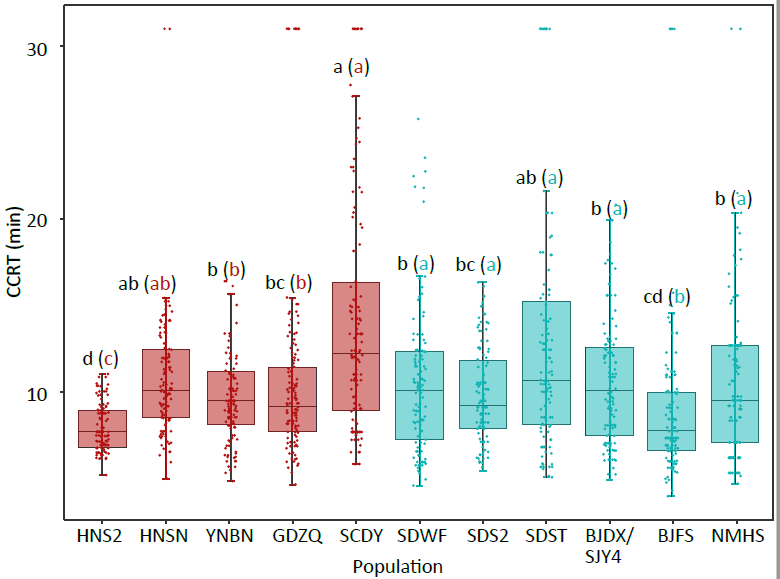


**Fig. S6** The CCRT values of different geographical populations of *Thrips palmi*. Box plot colors match genetic groups: red (Field group) and blue (Greenhouse group). The lowercase on the boxplot indicates the result of multiple comparisons between populations. Lowercase colors represent different groups: black (Both groups); red (Field group) and blue (Greenhouse group).


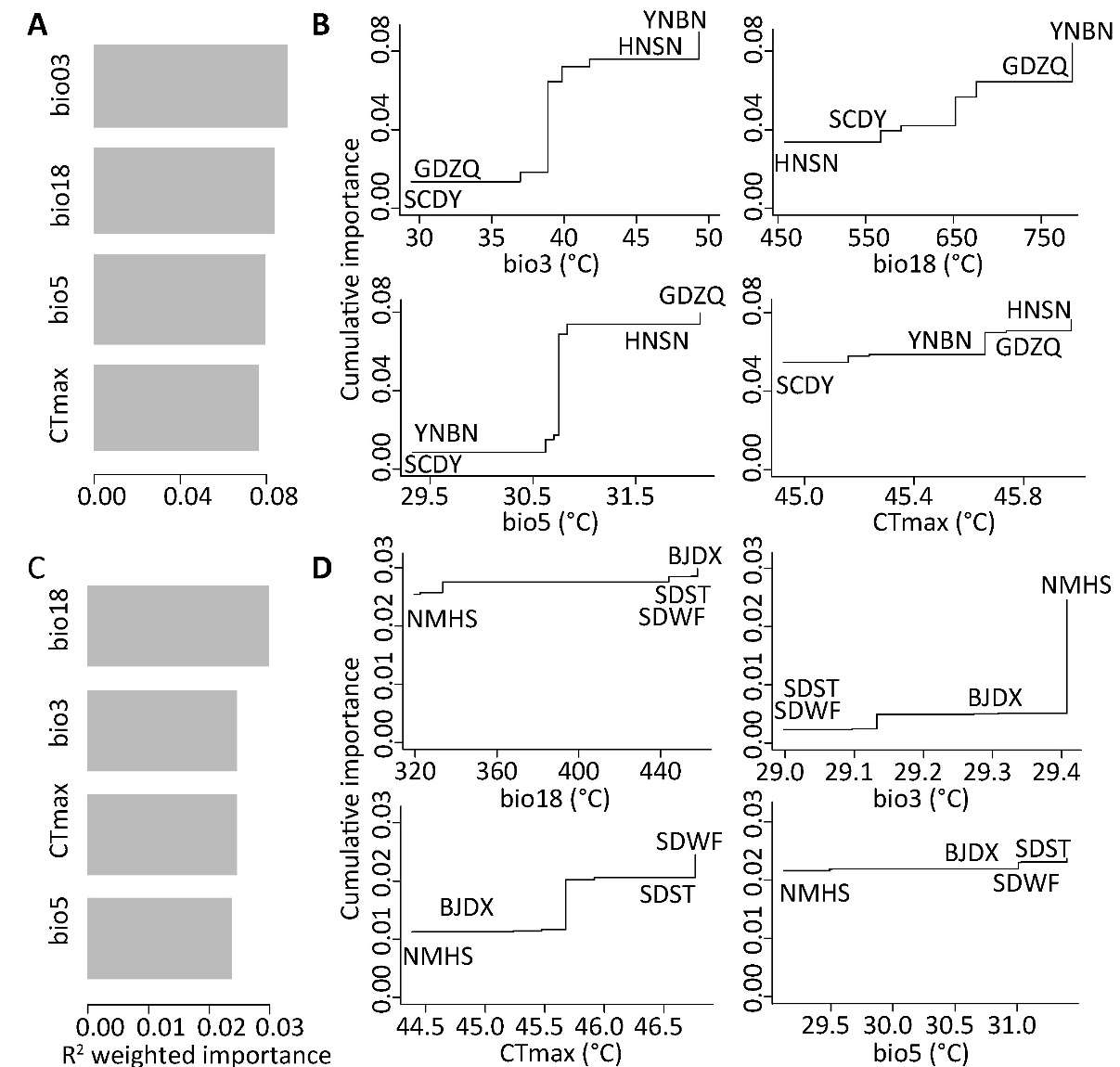


**Fig. S7** The importance of CT_max_ and climatic variables in the (A) Field and (C) Greenhouse groups. The turnover response of variables to adaptive genomic variation in (B) the Field group and (D) the Greenhouse group are presented.


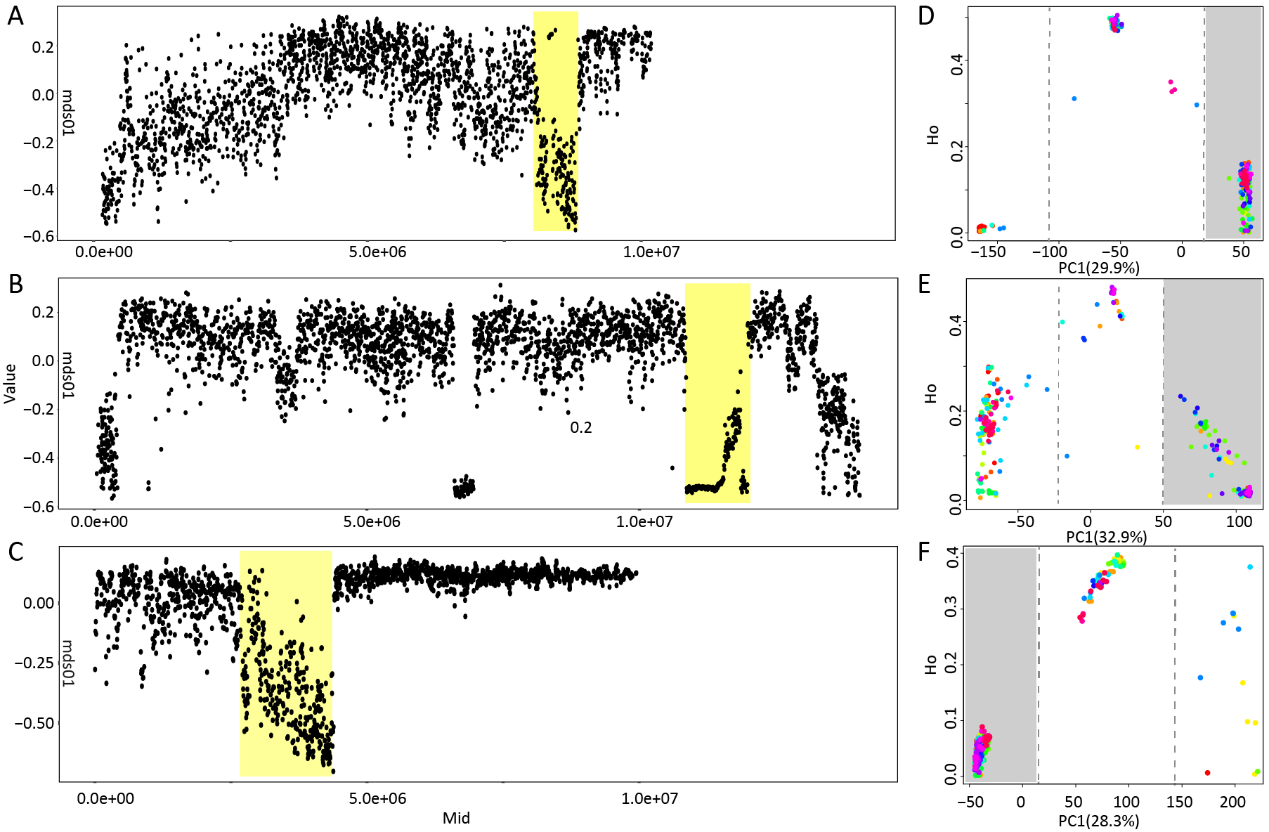


**Fig. S8** Structural variation of *Thrips palmi*. Figures A, B, C show local PCA analysis of chr3, chr5 and chr14. Dots represent multidimensional scaling (MDS) for 100 SNP windows along chromosomes; putative inversion regions are surrounded by a yellow box. Figures D, E, F show plots of the first component of a PCA and observed heterozygosity (*H*_O_) for the inversion regions of chr3, chr5 and chr14; two dotted lines separate all individuals into two homozygous genotypes (two flanks) and one heterozygous genotype (the middle area), and grey area indicates the homozygous genotype used for the estimation of pairwise linkage disequilibrium values in the lower triangle of the heatmap in Fig 3.


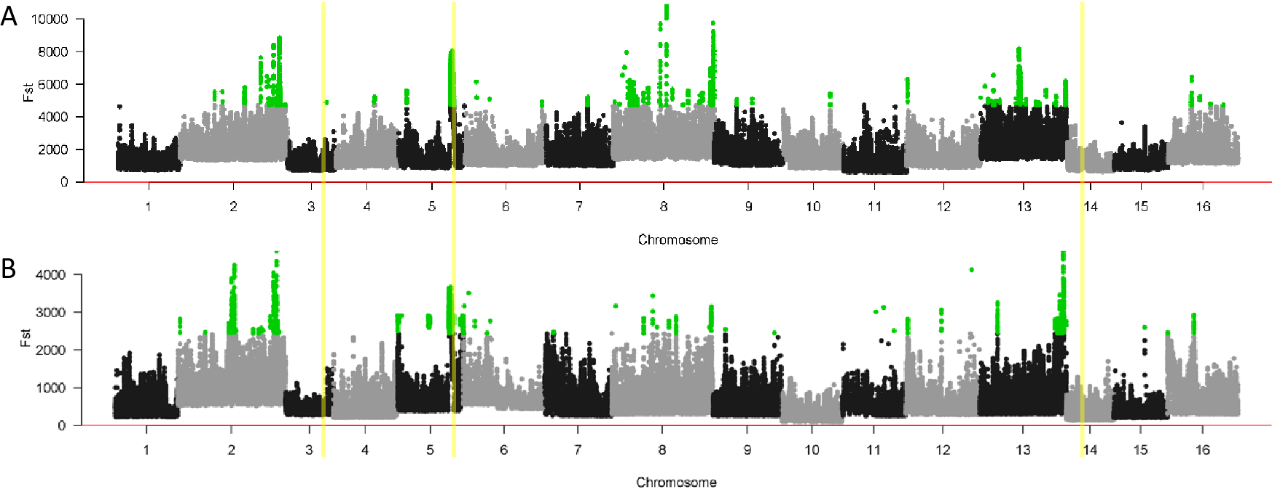


**Fig. S9** Genome-wide putative selective signatures in (A) Field group and (B) Greenhouse group. Manhattan plot for each window calculated by *F*_ST_ with k-nearest neighbor (kNN) algorithm. The top 1% outliers are marked by green points.


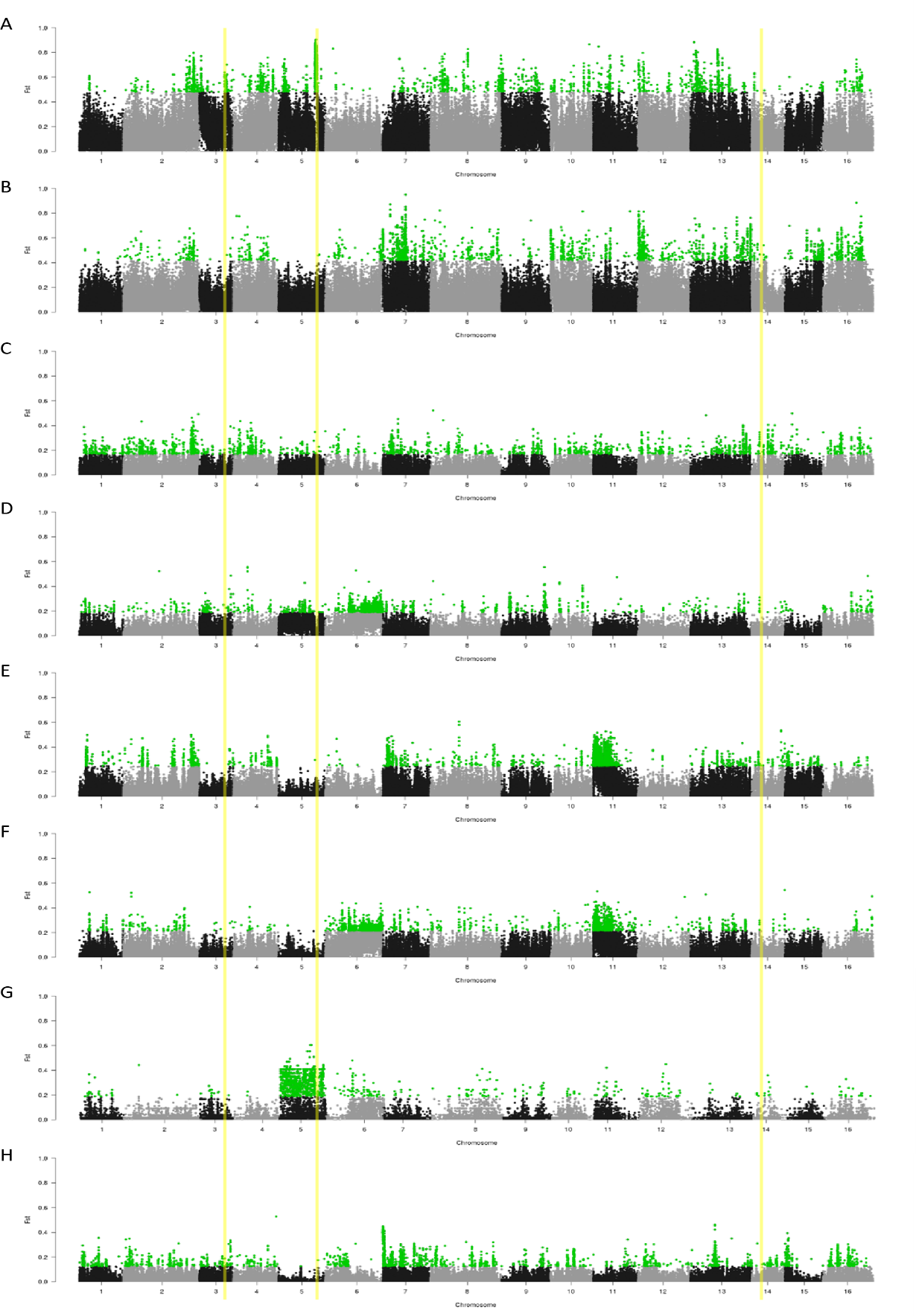


**Fig. S10** Manhattan plots of pairwise *F*_ST_ calculated using VCFtools based on all SNPs for different comparisons. The top 2% outliers are marked by green points. (A) HNSY vs SCDY population in Field group, (B) YNBN vs SCDY population in Field group, (C) BJDX vs SDS1 population in Greenhouse Greenhouse group, (D) NMHS vs SDS1 population in Greenhouse group, (E) BJDX vs SDS3 population in Greenhouse group, (F) NMHS vs SDS3 population in Greenhouse group, (G) Individuals with high and low CT_max_ values, (H) Laboratory-selected (SJY4) and control population (SJY1).


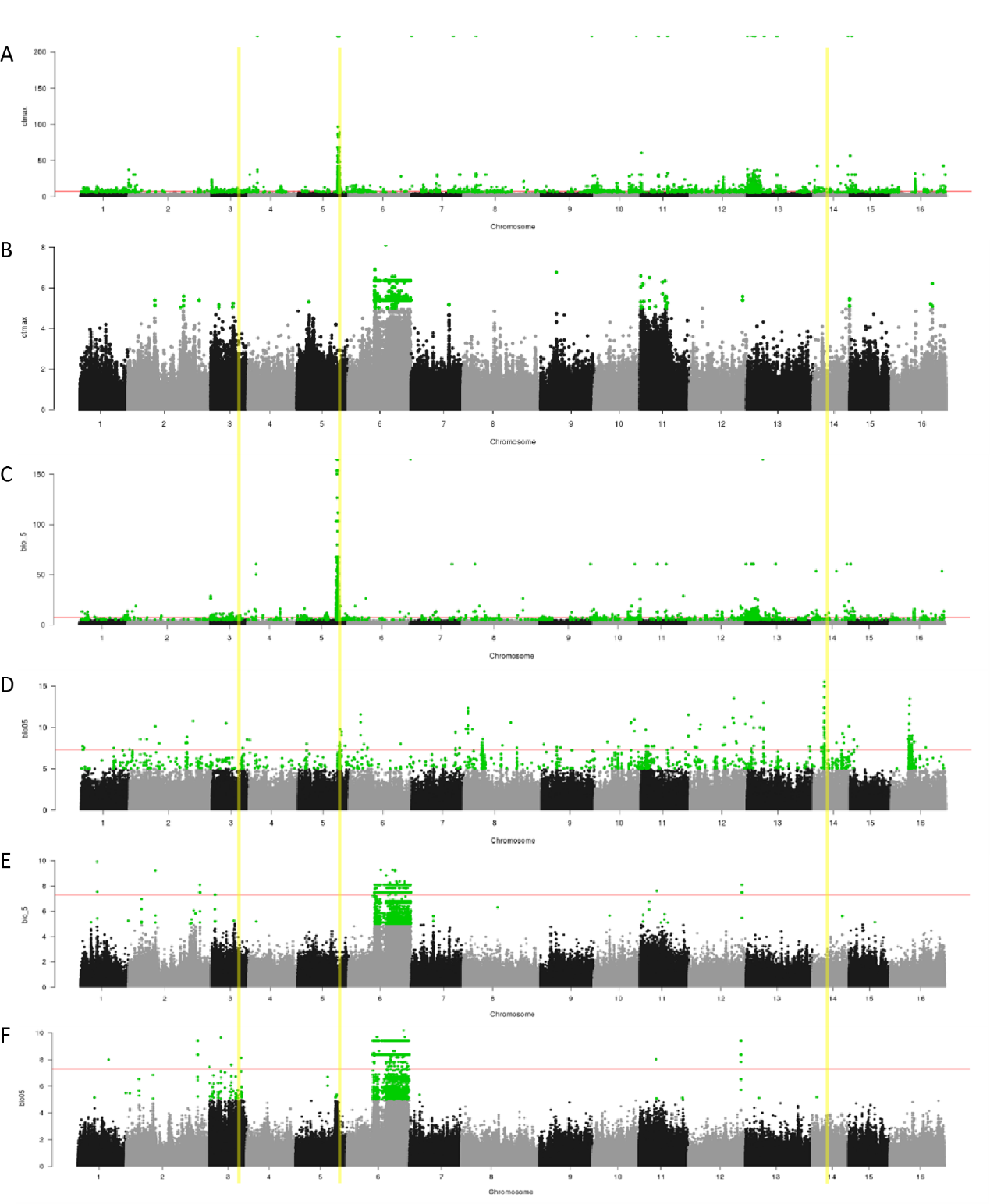
**Fig. S11** Manhattan plots depicting the results of a genome-wide association study of CT_max_ or bio05 using LFMM for different populations. The outliers with q-value<0.00001 (q-value<0.000001 was set in an association study of CT_max_ for four populations of Field groups) are marked by green points. (A) CT_max_ for four populations in the Field group, (B) CT_max_ for four populations in the Greenhouse group, (C) bio5 for four populations in the Field group, (D) bio5 for eight populations in the Field group, (E) bio5 for four populations in the Greenhouse group, (F) bio5 for six populations in the Greenhouse group.
